# Consequences of collagen induced inflammatory arthritis on circadian regulation of the gut microbiome

**DOI:** 10.1096/fj.202201728R

**Published:** 2022-12-15

**Authors:** Devin Amanda Simpkins, Polly Downton, Kathryn J. Gray, Suzanna H. Dickson, Robert J. Maidstone, Joanne E. Konkel, Matthew R. Hepworth, David W. Ray, David A. Bechtold, Julie Elizabeth Gibbs

**Affiliations:** ^1^ Centre for Biological Timing, Faculty of Biology Medicine and Health University of Manchester Manchester UK; ^2^ NIHR Oxford Biomedical Research Centre John Radcliffe Hospital Oxford UK; ^3^ Oxford Centre for Diabetes, Endocrinology and Metabolism University of Oxford Oxford UK; ^4^ Lydia Becker Institute for Immunology and Inflammation University of Manchester Manchester UK

**Keywords:** arthritis, circadian, colon, lactobacillus, microbiota

## Abstract

The gut microbiota is important for host health and immune system function. Moreover autoimmune diseases, such as rheumatoid arthritis, are associated with significant gut microbiota dysbiosis, although the causes and consequences of this are not fully understood. It has become clear that the composition and metabolic outputs of the microbiome exhibit robust 24 h oscillations, a result of daily variation in timing of food intake as well as rhythmic circadian clock function in the gut. Here, we report that experimental inflammatory arthritis leads to a re‐organization of circadian rhythmicity in both the gut and associated microbiome. Mice with collagen induced arthritis exhibited extensive changes in rhythmic gene expression in the colon, and reduced barrier integrity. Re‐modeling of the host gut circadian transcriptome was accompanied by significant alteration of the microbiota, including widespread loss of rhythmicity in symbiont species of *Lactobacillus*, and alteration in circulating microbial derived factors, such as tryptophan metabolites, which are associated with maintenance of barrier function and immune cell populations within the gut. These findings highlight that altered circadian rhythmicity during inflammatory disease contributes to dysregulation of gut integrity and microbiome function.

**Abbreviations**
BA: bile acid
CFA: Complete Freund's Adjuvant
CIA: collagen induced arthritis
EAE: experimental autoimmune encephalitis
IEC: intestinal epithelial cell
RA: rheumatoid arthritis
SCFA: short chain fatty acid
ZT: zeitgeber time

## 1 INTRODUCTION

The gut microbiome is composed of trillions of microorganisms (bacteria, archaea, viruses and eukaryotic microbes) which function to break down food in the gastro‐intestinal tract. This diverse community plays a critical role in shaping the host immune system, maintaining the integrity of the gut barrier and promoting immunological tolerance.^1^, ^2^ The composition of the bacterial community colonizing the intestinal tract is determined by multiple factors including diet and host immune responses. Conversely, the microbiota modulates host immunity through both direct host‐microbial interactions and via the action of microbial metabolites on host cells. Thus host‐microbial interactions are bi‐directional. Depletion of the microbiota (germ‐free animals or via antibiotic treatment) is associated with reduced susceptibility and attenuated symptoms of autoimmune diseases including experimental autoimmune encephalitis (EAE),^3^, ^4^ uveitis^5^ and inflammatory arthritis.^6^ Furthermore, there is a long‐established link between microbial dysbiosis and human inflammatory diseases such as inflammatory bowel disease^7^ and rheumatoid arthritis (RA).^8^ Several studies using single time point fecal sampling have identified perturbations in the gut microbiota in RA patients.^9^, ^10^, ^11^

Circadian (24 h) variation in the composition and activity of the microbiota adds a new dimension to our understanding of interactions between this community and host immunity.^12^ Recent studies have demonstrated that the composition, functional capacity, biogeographical localization and metabolic outputs of the gut microbiota varies over the course of the 24 h day.^13^, ^14^, ^15^, ^16^ Furthermore, daily rhythms in the microbiota are important for host health with loss of rhythmicity associated with altered intestinal innate and adaptive immunity^17^, ^18^, ^19^ and metabolic dysfunction.^20^ Mechanisms driving daily oscillations in the microbiome are not fully understood, but these rhythms are clearly influenced by dietary composition^14^ and host behavioral rhythmicity, including timing of food intake,^13^, ^14^ also by the host circadian clock acting through neural and humoral outputs.^13^, ^16^ We have identified rhythmic secretion of immunoglobulin A (IgA) as an important mechanism driving oscillations in the abundance of a number of commensal bacteria^21^ and more recently a critical role for the intestinal epithelial cell (IEC) clock in driving the rhythmic microbiome has been demonstrated.^22^

The circadian clock regulates a huge range of physiological processes via temporal control of cellular function. The nature of this regulation is cell, tissue and organ dependent, with 43% of all protein coding genes showing circadian rhythms in transcription somewhere in the body.^23^ In brief, 24 h rhythms in cellular functions are regulated by a molecular transcriptional‐translational feedback system involving a small group of core clock proteins (reviewed in Ref. [24]). BMAL and CLOCK drive expression of *Period* and *Cryptochrome*. The protein products PERIOD and CRYPTOCHROME in turn inhibit CLOCK/BMAL promoted gene expression. The whole process takes approximately 24 h. Further proteins, REV‐ERBs and RORs form an auxiliary loop which contributes to the robustness of clock function.^25^, ^26^ Disruption of the clock through gene knockout or environmental manipulation leads to disordered rhythms in the microbiota, although this is to some extent secondary to altered feeding patterns.^10^, ^15^

Our recent work illustrates that in the setting of inflammatory arthritis, clocks within resident and recruited immune cells show reduced amplitude at the site of inflammation^27^ which corresponds to our understanding of the effect of inflammatory mediators on the core clockwork machinery, acting on REV‐ERB expression in particular.^28^ In addition, we have recently identified that chronic joint inflammation leads to temporal re‐organization of clock‐regulated processes at distal sites, including liver and muscle.^29^ We now want to assess how chronic inflammatory arthritis affects the core clock and clock regulated processes within the gut and ask what the consequences are of any change for the microbiota and its metabolic outputs.

## 2 MATERIALS AND METHODS

### 2.1 Animal studies

All experimental procedures were carried out under the UK Animals (Scientific Procedures) Act 1986 and were subject to local ethical review from the University of Manchester Animal Welfare and Ethical Review Board. All animals were housed in isolated ventilated cages under a 12:12 light:dark cycle, where zeitgeber time 0 (ZT0) refers to lights on and ZT12 refers to lights off. Animals had *ad libitum* access to normal chow, and naïve and arthritic mice were co‐housed.

### 2.2 Collagen induced arthritis (CIA)

Male DBA/1 mice (Envigo, 9–10 weeks) were immunized (day 0) with intradermal injections (50 μl in two separate sites above the tail) of bovine type II collagen (MD biosciences, Switzerland) emulsified in Complete Freund's adjuvant (CFA, MD biosciences) at 1 mg/ml. A boost of bovine type II collagen (diluted to 1 mg/ml in saline) was administered intraperitoneally on day 21. Both the immunization and the boost were administered in the mid‐light phase (ZT6). Disease developed from day 18 (peak incidence at day 23 – day 29) and animals were scored for disease severity on a scale of 0–4 per limb.^30^ Colonic tissue, whole caecum, terminal blood and fecal samples were collected at ZT0, ZT4, ZT8, ZT12, ZT16 or ZT20, 7 days after the development of disease symptoms in CIA mice or at matched time points in naïve co‐housed mice.

### 2.3 RNA sequencing

A 5 mm section of mid‐colon was snap frozen and prepared for RNA Seq. Tissue was transferred to a Lysing matrix D tube containing Trizol. Tissue was homogenized using a Bead Mill homogenizer. RNA was extracted using chloroform then precipitated using isopropanol. After washing with 75% ethanol, the RNA pellet was re‐suspended in RNase free water (50 μg/ml). Sequencing library preparation and sequencing was performed by the University of Manchester Genomic Technologies Core Facility. Sample quality was determined using a 2200 TapeStation (Agilent technologies). Libraries were generated using the TruSeq Stranded mRNA assay (Illumina Inc) according to the manufacturer's protocol. The multiplexed libraries were analyzed by paired‐end sequencing on a HiSeq 4000 instrument (76 + 76 cycles, plus indices), then demultiplexed and converted using bcl2fastq software (v2.17.1.14, Illumina). Adaptors were removed and ends trimmed using Trimmomatic (v0.36), then reads were mapped against the mouse genome (mm10/GRCm38) using STAR (v2.5.3). Reads were counted, normalized and annotated in R using the Rsubread (v1.28.1), edgeR (v3.30.3) and biomaRt (v2.44.0) packages, respectively.

Differential expression analysis was run in RStudio using edgeR (v3.28.1) (and then additional packages listed in Table S1) using quasi‐likelihood F (QLF) test. Genes were considered to be differentially expressed (DE) if false discovery rate (FDR) was <0.05 and log fold change of <−2 or <2. Rhythmicity analysis was completed in R studio using compareRhythms^31^ whereby differentially rhythmic (DiffR) genes were identified by comparing rhythmicity patterns between two treatments to classify genes as arrhythmic or as having a “gain”, “loss”, “change” or the “same” rhythmic pattern. Pathway analysis of selected gene lists used the Enrichr web tool^32^, ^33^ to detect significantly enriched pathways within the KEGG 2019 mouse database.

### 2.4 IgA and albumin ELISA

Fecal pellets were re‐suspended in PBS (1 mg/ml) and homogenized (Bead Mill 24 homogenizer, FisherBrand). Samples were then centrifuged 3 times at 200*g*, 8000*g* and 10 000*g* for 5 min each, removing the pellet each time. The final supernatant was retrieved and stored at −80°C until use. Total concentration of fecal IgA was determined by ELISA using a commercial kit (Bethyl laboratories, USA) as per manufacturer's instructions. Briefly, plates were coated with purified anti‐mouse IgA antibody and incubated at RT for 30 min. After 5× washes (PBS‐0.05% tween 20) wells were blocked with 1% bovine serum albumin (Sigma‐Aldrich, St Louis, MO) and incubated at RT for 30 min. Plates were washed and then duplicate standards or stool samples (diluted 1:8) were added to the plates and incubated at RT for 1 h. After further washes the plates were incubated with HRP conjugated goat anti‐mouse IgA detection antibody (0.1 mg/ml diluted to 1:30 000) at RT for 1 h. Plates were washed and 50 μl of substrate reagent (R&D systems) was allowed to develop. The reaction was stopped by adding 100 μl of 0.16 M H_2_S0_4_ to each well. The absorbance of the plates was detected at 450 nm (Promega GloMax).

Total concentration of fecal albumin was determined by ELISA using a commercial kit (Bethyl laboratories, USA) as per manufacturer's instructions. Briefly, plates were coated with purified goat anti‐mouse albumin antibody and incubated at RT for 30 min. After washing and blocking (described above) duplicate standards or stool samples were added to the plate and incubated at RT for 1 h. After washes the plates were incubated with HRP conjugated goat anti‐mouse albumin detection antibody (0.1 mg/ml diluted to 1:15 000 in 1 × PBS‐tween 20) at RT for 1 h.

### 2.5 Fluorescein isothiocyanate (FITC)‐dextran assays

FITC‐dextran assays were utilized to assess gut barrier permeability prior to induction of arthritis (day ‐1) and during the symptomatic stage (day 27 onwards). For controls, a naïve group of mice was assayed across both timepoints. Animals were fasted for 3 h, then gavaged at ZT0 with 150 μl of 40 mg/ml 4 kDa FITC‐dextran (Sigma) in sterile water. After 4–5 h a blood sample (50 μl) was collected via the tail vein into lithium heparin coated collection tubes (Greiner). Samples were centrifuged (10 000*g*, 10 min) and the resultant plasma was diluted 1 in 4. 100 μl of each sample was added to a 96 well plate to assess fluorescence (Promega GloMax plate reader, 485 nm excitation/528 nm emission). Values were interpolated based on the standard curve created using FITC‐dextran diluted in sterile water.

### 2.6 Histology

Colons were excised from animals and prepared for histology.^34^ In brief, colons were flushed and fixed with modified Bouin's solution (50% ethanol, 5% acetic acid in distilled water) using a syringe and gavage needle. The intestinal section was then opened longitudinally, rinsed briefly in PBS and rolled from the proximal end into a “swiss roll”, before transferring to a tissue‐processing cassette. The sample was placed in 10% buffered formalin overnight (RT) and then dehydrated through ethanol and xylene before being embedded in paraffin blocks and sectioned at 5 μm thickness (Leica RM2255 microtome). Sections were mounted onto X‐tra™ Adhesive slides (Leica). Hematoxylin and eosin staining was performed using standard protocols. Images were taken using the 3D‐Histech Pannoramic‐250 microscope slide‐scanner. Histology images were processed using the opensource software FIJI.^35^

### 2.7 Flow cytometry (lamina propria)

Excised colons were cleared of fecal matter, cut longitudinally and rinsed in ice cold PBS before transfer to PBS containing 2% FBS on ice. Tissue was subsequently transferred into PBS containing 2 mM EDTA and 1 mM DTT then incubated for 15 min at 37°C, shaking at 180 rpm, then filtered (70 μm). The tissue was incubated in a digestion enzyme cocktail (Collagenase V (0.85 mg/ml); Collagenase D (1.25 mg/ml); Dispase II (1 mg/ml) and DNase (30 μg/ml)) for 25 min, before vortexing, filtering (40 μm) and re‐suspending cells. Cell staining was carried out in a 96‐well V‐bottomed plate. After live/dead staining (LIVE/DEAD fixable blue dead stain kit, ThermoFisher Scientific) and blocking (1:100 anti mouse CD16/CD32, Fisher Scientific, UK) cells were stained with a panel of antibodies (Table S2). All extracellular antibodies were utilized at 1:200 and intracellular at 1:100. Amine Reactive Compensation beads (Thermo Fisher Scientific) were utilized with the live/dead stain for compensation controls. One Comp eBeads (ThermoFisher Scientific) were utilized to produce single stained compensation controls. Samples were analyzed on a BD LSR Fortessa Flow cytometer and data collected using FACSDiva software. Data analysis was performed using Flow Jo Version 10.7.1.

### 2.8 16S rRNA sequencing

Fecal samples were collected from the terminal end of the colon (2–3 pellets/mouse), frozen and stored at −80°C. The samples were collected at 6 time points: ZT0, ZT4, ZT8, ZT12, ZT16 and ZT20. Bacterial DNA was isolated using the DNeasy PowerSoil kit (Qiagen) according to manufacturer's instructions. DNA was eluted in 10 mM Tris–HCL buffer pH 8.5. 20 μl of PCR product (1 ng/μl) was sequenced by the University of Liverpool Centre for Genomic Research, using the primers which spanned the variable region 2 (V4) of the 16S rRNA gene:

Forward: 5′‐ACACTCTTTCCCTACACGACGCTCTTCCGATCTNNNNNGTGCCAGCMGCCGCGGTAA‐3′.

Reverse: 5′GTGACTGGAGTTCAGACGTGTGCTCTTCCGATCTGGACTACHVGGGTWTCTAAT‐3′.

PCR products were then sequenced with the Illumina MiSeq platform, using 2 × 250 paired‐end sequencing. This generated 200 000–400 000 reads per sample. Fastq files were then trimmed and reads shorter than 20 bp were removed.

The reads were then processed using Centaurus Galaxy Server, Galaxy for the University of Manchester at the Faculty of Biology, Medicine and Health (FBMH). Briefly, reads were paired and taxonomic information was mapped to operational taxonomic unit (OTU) using the reference database Silva. Data was visualized using RStudio (http://www.rstudio.com/) and additional packages (Table S1).

### 2.9 Metabolomics

#### 2.9.1 Short chain fatty acids (SCFA)

Caecal samples were snap frozen at the time of collection. Levels of short chain fatty acids (SCFAs) were quantified by Metabolon (Durhan, NC, USA) using an Agilent 1290/AB Sciex QTrap 5500 LC–MS/MS system equipped with a C18 reversed phase UHPLC column (Metabolon method TAM135). The tissue was spiked with stable labeled internal standards and the following quantified: acetic acid (C2), propionic acid (C3), isobutyric acid (C4), butyric acid (C4), 2‐methyl‐butyric acid (C5), isovaleric acid (C5), valeric acid (C5) and caproic acid (Hexanoic acid, C6).

#### 2.9.2 Global un‐targeted metabolomics

Terminal blood was collected in EDTA‐coated tubes and stored on ice prior to centrifugation at 3000*g* for 10 min at 4°C. Plasma was transferred to a cryovial and flash frozen in liquid nitrogen. Global metabolite profiling of plasma samples was performed by Metabolon (Durham, NC, USA). Samples were analyzed using the HD4 platform, which uses ultrahigh performance liquid chromatography‐tandem mass spectrometry (UPLC‐MS/MS) methods for metabolite detection. Peaks were quantified by area under the curve, and normalized to set metabolite median value to one.

### 2.10 Data analysis

Differential rhythmicity analysis was performed using compareRhythms R package (v0.99.0).^29^, ^31^ A model selection approach was used with genes being assigned to either arrhythmic, gain of rhythm, loss of rhythm, same rhythm in both, or a change in rhythm. A probability of being in a category of at least 0.6 was required for assignment. Rhythmicity analysis was run using the JTK‐cycle functionality of MetaCycle (v1.2.0), with period length fixed to 24 h. Microbes and metabolites were considered to oscillate if the JTK‐cycle adj*p* < .05. Statistical analysis was performed using GraphPad Prism version 7.0. Unless stated otherwise, data is presented as mean ± standard error of the mean (SEM). Comparisons between two groups were made using two‐tailed Student's *t* tests. Comparisons between multiple groups were made using one‐way ANOVAs and appropriate post‐hoc tests with correction for multiple comparisons as defined in the figures. Where two variables (e.g., time‐of‐day and treatment) were included, two‐Way ANOVAs were utilized with appropriate post‐hoc tests with correction for multiple comparisons as defined in the figures.

## 3 RESULTS

### 3.1 Rhythmic transcriptional programs within the colon become re‐organized in CIA

**FIGURE 1:**
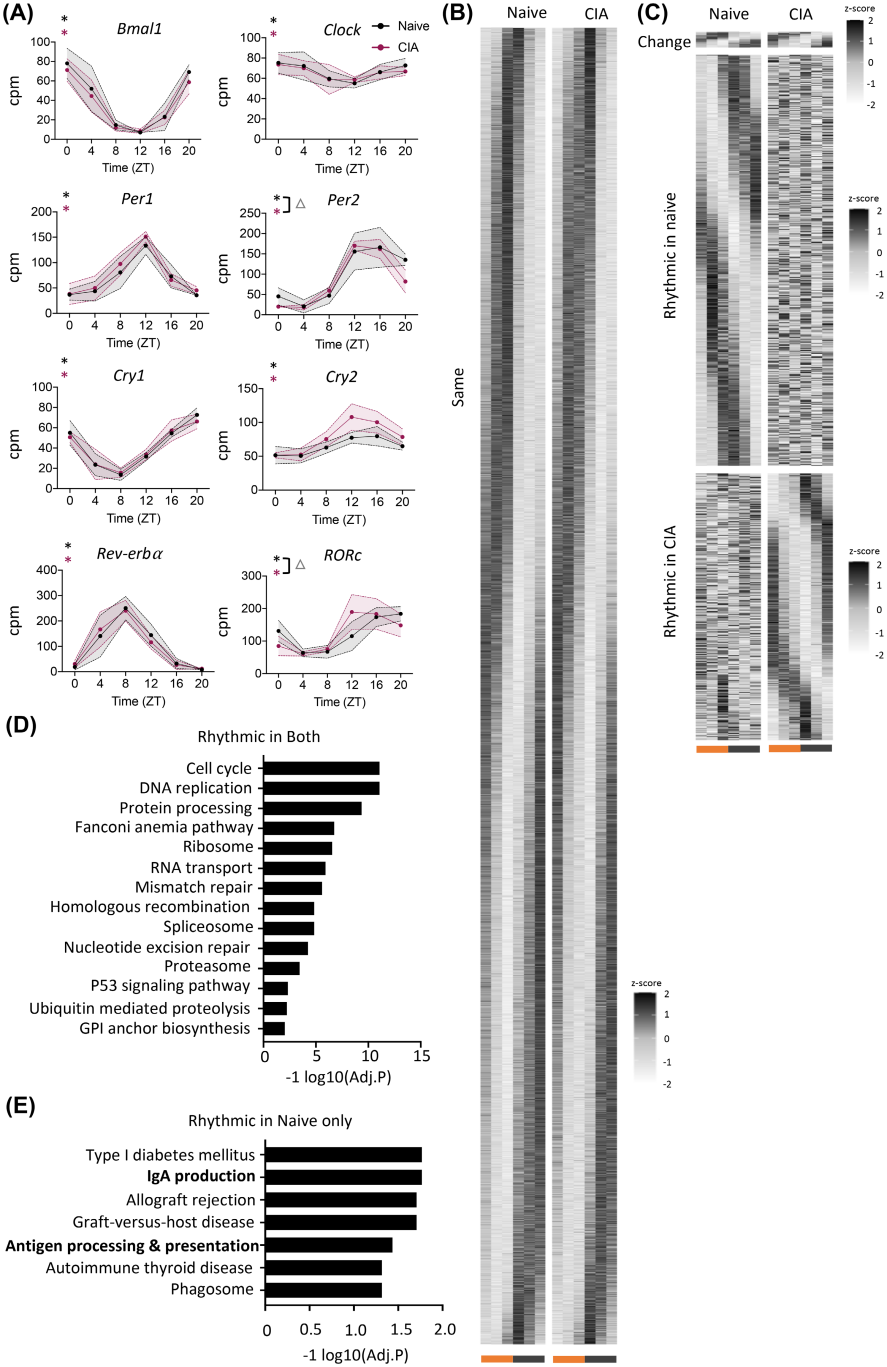
Temporal gene expression in colonic tissue from CIA and naïve animals. (A) Expression of core clock genes in naïve and arthritic colonic tissue measured during RNA sequencing, *n* = 5/time point, values are mean ± SD. Compare rhythms was used to define rhythmicity where * denotes significantly rhythmic transcripts and △ denotes a change in amplitude or phase between treatments (naïve versus CIA). (B and C) Heatmaps illustrating the temporal organization of transcripts across the 24 h day (orange: ZT0, ZT4 and ZT8 and black: ZT12, ZT16, ZT20), *n* = 5/time point. Transcripts are grouped as same (no change in rhythmicity); change (remains rhythmic but change in peak or phase); rhythmic in naïve (but not in CIA); and rhythmic in CIA (but not in naïve). Pathway analysis (KEGG mouse 2019, *p*adj < .01) of transcripts that are rhythmic in (D) both naïve and arthritic colon (same) and (E) naïve tissue only.

To assess the consequences of chronic inflammatory arthritis on the rhythmic transcriptome of the gut, RNA Seq was performed on whole colonic tissue samples collected around the clock from naïve and arthritic (CIA) mice. CompareRhythms^31^ was used to assess transcript rhythmicity between naïve and arthritic mice. Examination of the temporal expression of genes encoding the core clockwork machinery confirms that the gut clock remains predominantly intact in inflammatory arthritis (Figure 1A), although minor changes were observed in the rhythmic expression of *Per2* and *RORc*. We found that 6901 transcripts (55.7% of all detected) were identified as rhythmic in colonic tissue from naïve animals (Figure 1B,C). These mapped onto a wide range of pathways including cell cycle, DNA replication and protein processing (Figure 1D). CIA caused a re‐organization of these daily rhythms, with loss (rhythmic in naïve only: 604 transcripts) and gain (rhythmic in CIA only: 399 transcripts) of rhythms in addition to change in rhythmicity (change: 43 transcripts) (Figure 1C). Pathway analyses of transcripts which were only rhythmic in naïve animals/colon highlighted immune related processes including the intestinal immune network for IgA production and antigen processing and presentation (Figure 1E).

### 3.2 CIA alters the expression of genes in the IgA production pathway and tight junction formation

**FIGURE 2:**
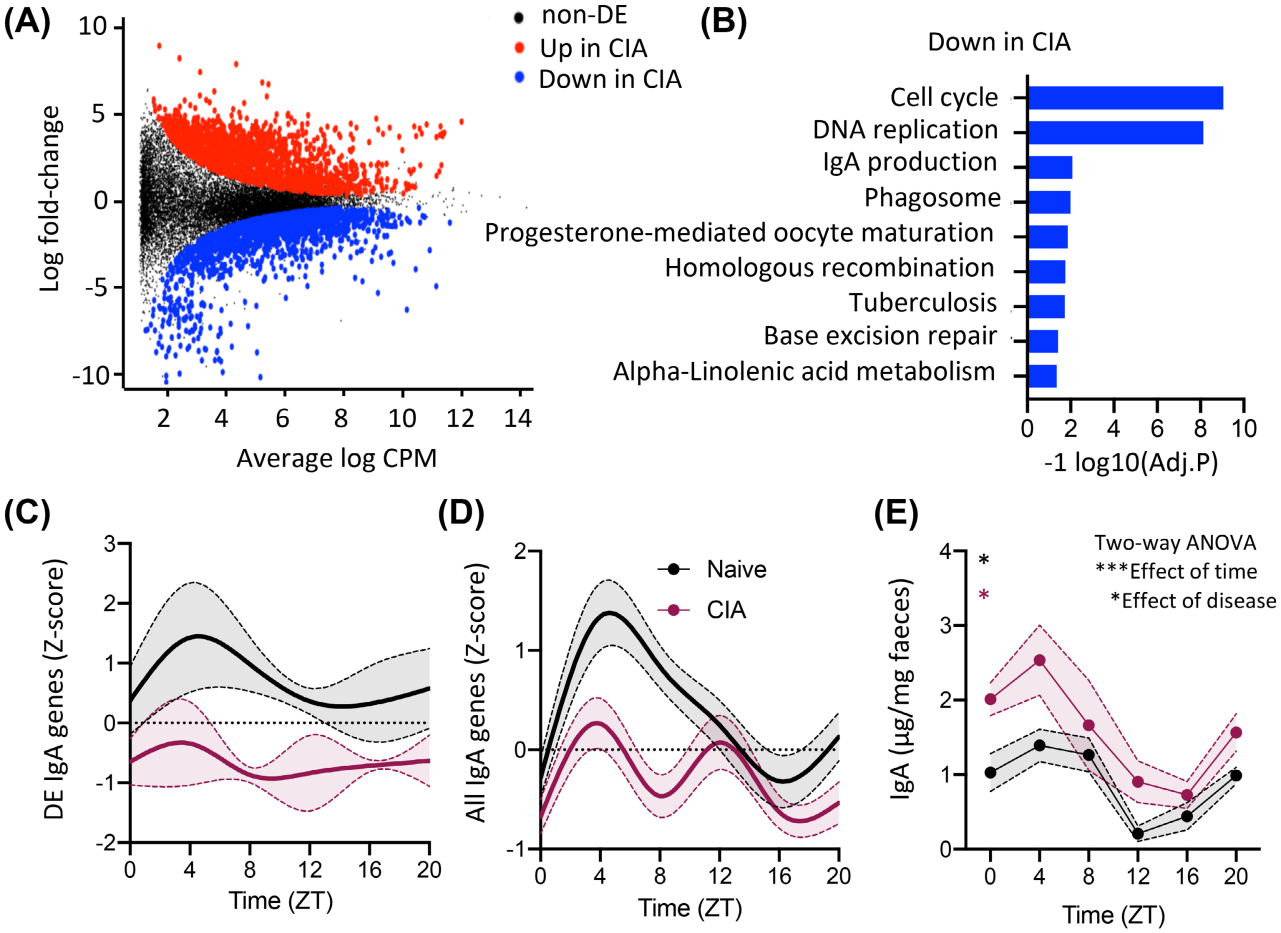
Rhythmic IgA production in the setting of arthritis. (A) Volcano plot illustrating differentially expressed (DE) transcripts (FDR <0.05) in colonic tissue from naïve (*n* = 30) and arthritic (*n* = 30) mice. (B) Pathway analysis (KEGG mouse 2019, *p*adj < .05) of transcripts that are down in CIA compared to naïve tissue. Temporal expression of (C) differentially expressed (DE) genes and (D) all genes in the IgA pathway in naïve and arthritic colon, *n* = 5/time point, values are mean ± SD. (E) IgA levels in fecal pellets collected longitudinally over 24 h from naïve and arthritic mice (5 mice per treatment group), values are mean ± SEM, * denotes significant 24 h rhythms (assessed using Compare Rhythms). Two‐way ANOVA revealed a significant effect of time (*P* < 0.005) and disease (*P* < 0.05) on fecal IgA levels.

In addition to addressing the consequences of arthritis on the rhythmicity of the gut transcriptome, we also compared differentially expressed (DE) transcripts between treatment groups. Examination of DE transcripts within the colonic tissue of naïve and arthritic mice (pooling all time points, *n* = 30/treatment) revealed a profound change in transcriptional profile (Figure 2A). Pathway analyses revealed significant down‐regulation of pathways included in the cell cycle process, DNA replication and IgA production (Figure 2B). Given the role for IgA in maintenance of microbial diversity^34^ and rhythmicity^21^ we explored this pathway in further detail. Of all genes assigned to this pathway (43 genes) 8 exhibited significant down regulation (Figure 2C,D). Given that secretion of IgA exhibits 24 h oscillations,^21^ we went on to determine if these transcriptional changes in the colon resulted in a change in rhythmic IgA production. Assays were performed on fecal samples collected from a separate cohort of animals over a 24 h period (Figure 2E). In the setting of inflammatory arthritis, rhythms in fecal IgA concentrations persisted, but unexpectedly IgA concentrations were elevated across the 24 h day (two‐way repeated measures ANOVA: effect of time *p* = .0011; effect of treatment *p* = .0143; interaction NS).

**FIGURE 3:**
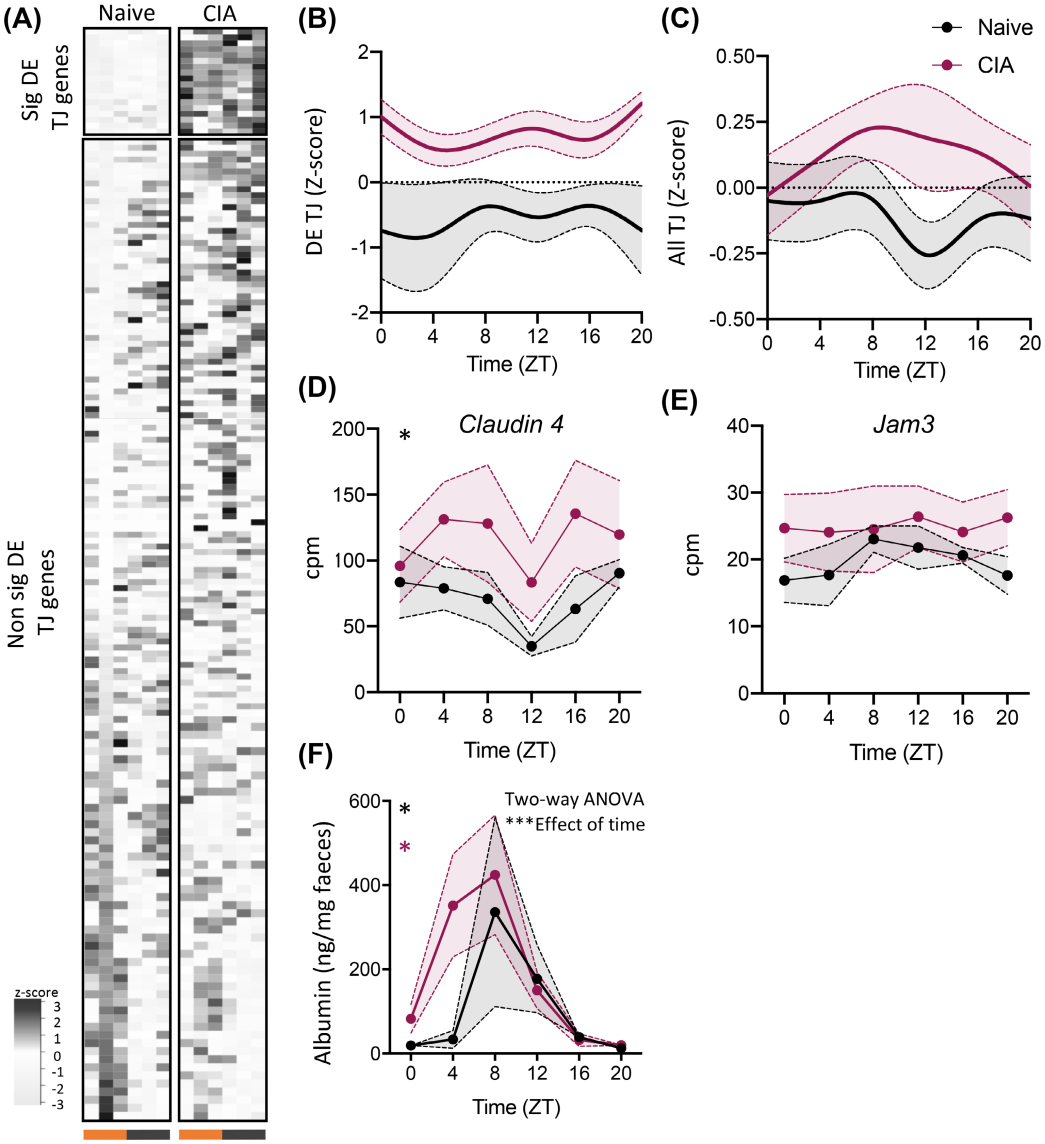
CIA alters colonic expression of tight junction genes and barrier permeability. (A) Heat maps illustrating the temporal organization of transcripts from the tight junction pathway across the 24 h day (orange: ZT0, 4 and 8 and black: ZT12, ZT16, ZT20), *n* = 5/time point. Transcripts which exhibited significant differential expression between treatment groups are highlighted in the upper portion. Temporal expression of (B) differentially expressed (DE) genes and (C) all genes in the tight junction genes pathway in naïve and arthritic colon, *n* = 5/time point, values are mean ± SD. Temporal expression of (D) *Claudin 4* and (E) *Jam3* in naïve and arthritic colon, *n* = 5/time point, values are mean ± SD, * denotes significant 24 h rhythms (assessed using Compare Rhythms). (F) Albumin levels in fecal pellets collected longitudinally over 24 h from naïve and arthritic mice (6 mice per treatment group), values are mean ± SEM, * denotes significant 24 h rhythms (assessed using Compare Rhythms). Two‐way ANOVA revealed a significant effect of time (*P* < 0.005) on fecal albumin levels.

Further analysis of DE transcripts revealed that CIA was associated with an up regulation of pathways involved in barrier integrity including adherens junction, tight junction and cell adhesion molecules (Figure 3A and Figure S1). There was generalized up‐regulation across the tight junction pathway irrespective of time. Genes of the tight junction pathway which exhibited significant upregulation (Figure 3B,C) included *Claudin 4* and *Jam3* (Figure 3D,E). To determine whether these changes related to potential alterations in barrier function in arthritic mice we quantified albumin content in fecal samples across the 24 h day.^36^ Results revealed a significant diurnal rhythm in fecal albumin which peaked at ZT8 in naïve animals, and was significantly increased during the day (ZT0‐8) in arthritic animals. To further corroborate this observation, FITC‐dextran assays were performed in an additional cohort of mice as a secondary measure of gut barrier permeability (Figure S2). Results demonstrated increased barrier permeability during the early rest phase in animals showing disease symptoms (day 27 onwards). Of note, no significant change in gut permeability to FITC‐dextran was observed in mice after the collagen boost and prior to the development of symptoms (day 22 – data not shown). Together these functional assays report a more leaky gut barrier in the daytime in arthritic animals, and data from the transcriptomics imply failed attempts to strengthen the epithelial barrier in the CIA mice.

### 3.3 CIA is not associated with inflammatory mediators within the colon

Our data reveals changes in gene expression and within the integrity of the gut barrier in the setting of established CIA. A number of approaches were taken to ensure these were not a consequence of local inflammation within the colon. We first applied xCell,^37^ a gene signature‐based bioinformatics method, to infer immune and stromal cell subtypes from RNA Seq datasets, which revealed comparable immune cell subsets between treatment groups (Figure S2). In keeping with this, analysis of immune cell populations within the colonic lamina propria by flow cytometry (gating strategies Figure S4A) revealed no difference in the total number of lymphocytes nor proportions of myeloid cells, B cells or T cell populations or eosinophils (Figure S4B). Finally, histological sections of colon prepared from naïve and arthritic animals were compared and villi thickness and numbers of inflammatory foci quantified (Figure S5A–D). These parameters remained consistent between naïve and arthritic mice. Taken together, these data indicate that in our arthritic animals there was no localized inflammation within the gut.

### 3.4 CIA is associated with alterations to the rhythmic gut microbiota

**FIGURE 4:**
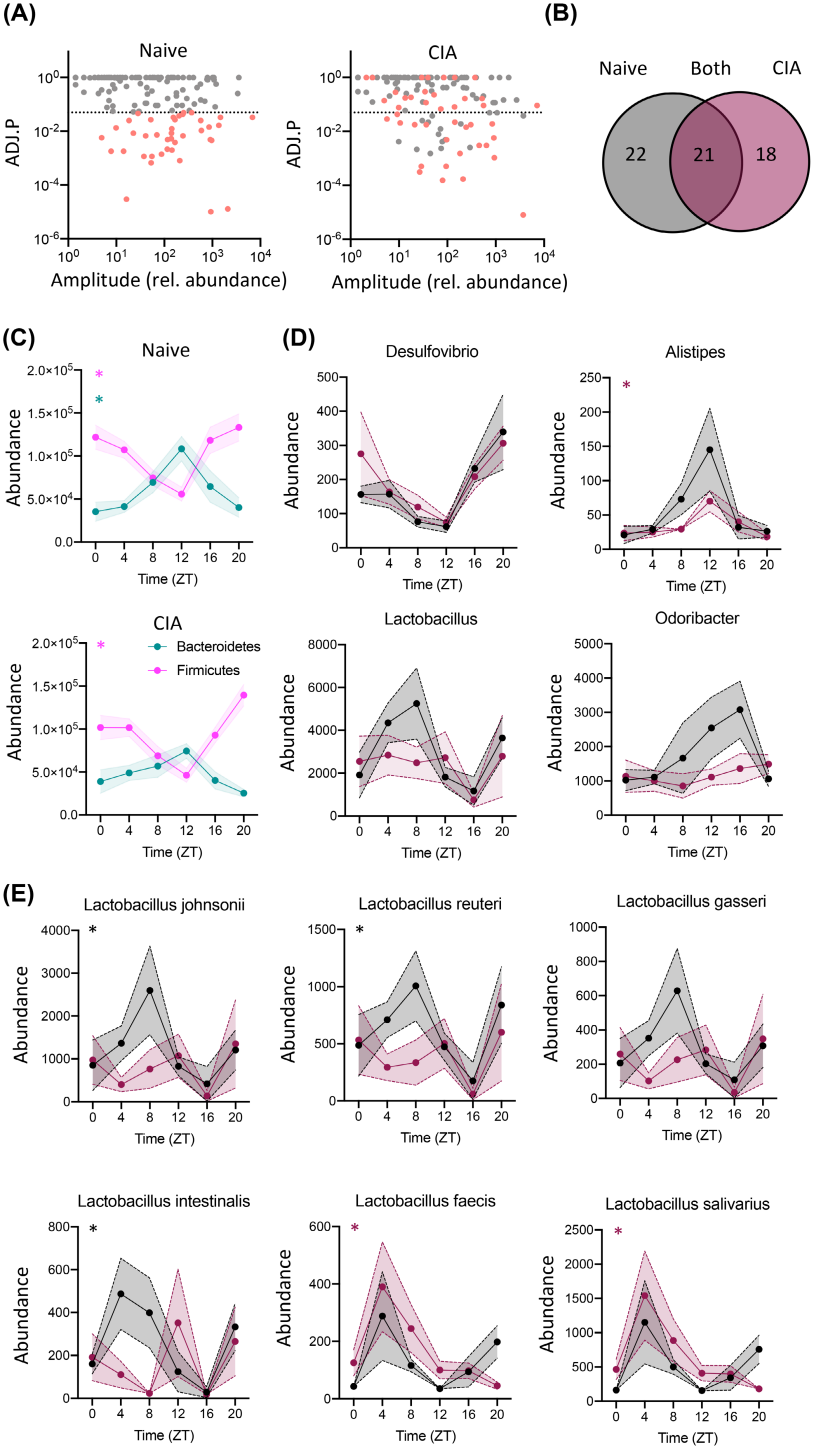
CIA is associated with altered daily rhythms in the gut microbiota in a taxa specific manner. (A) Abundance and rhythmicity (JTK_Cycle analysis) of the OTUs detected in each condition. OTUs which have are significantly rhythmic in naïve animals (*p*adj < .05) are highlighted in pink in both graphs. (B) Numbers of significantly rhythmic (*p*adj < .05) OTUs in each treatment group. (C) Abundance of the two dominant phyla bacteroidetes and firmicutes across the circadian day in naïve and CIA animals (*n* = 5/time point), values are mean ± SEM, * denotes significant 24 h rhythms (*p*adj < .05) (assessed using JTK_Cycle analysis). (D) Abundance of 4 bacterial genera across the circadian day in naïve and CIA animals (*n* = 5/time point), values are mean ± SEM, * denotes significant 24 h rhythms (*p*adj < .05) (assessed using JTK_Cycle analysis). (E) Abundance of 6 species of lactobacillus across the circadian day in naïve and CIA animals (*n* = 5/time point), values are mean ± SEM, * denotes significant 24 h rhythms (*p*adj < .05) (assessed using JTK_Cycle analysis).

Transcriptomic analyses highlighted changes within the colon of CIA affected mice which may alter the mechanisms by which the host manages the microbiota. To address this possibility, the bacterial composition of the gut microbiota was assessed via 16S rRNA of fecal samples collected at the time of cull from CIA and naïve mice. The numbers of OTU detected in each sample ranged from 90 597 to 221 575. Taxonomic analysis identified 163 species across 8 phyla (Figure S6A). Assessment of species diversity using the Shannon index (Figure S6B) revealed no significant differences between naïve and arthritic animals. Similarly, beta‐diversity analysis (Bray‐Curtis) revealed no significant difference in diversity between the two treatments (data not shown). We next considered how CIA affects rhythmicity of the gut microbiota. Assessment of OTU rhythmicity revealed that a significant proportion of the microbial community exhibits 24 h rhythmicity within both naïve (26%) and arthritic animals (24%), but there is significant temporal re‐organization in the setting of arthritis as only approximately 46% of rhythmic OTUs are shared between conditions (Figure 4A,B). Our earlier work has established these mice (both CIA and naïve) exhibit robust diurnal rhythms in locomotor and feeding behavior.^29^ CIA does not cause a change in total daily food intake or timing of food intake, with the majority of the diet (~80%) consumed during the night. Thus, the observed changes in microbial profile and rhythmicity are not secondary to alterations in feeding/activity patterns.

To explore this temporal remodeling of the microbiota further, we analyzed the impact of CIA at the bacterial phyla, genera and species level identified by our sequencing (Figure 4C,D). As expected^38^ the most abundant phyla, the *Firmicutes* and *Bacteroidetes*, exhibited diurnal rhythmicity in naïve animals and were in antiphase to one another. Of note rhythmicity in the *Bacteroidetes* was dampened in the setting of CIA (Figure 4C). Further taxonomic profiling at the genera level revealed reductions in peak abundance in several microbes including *Odoribacter*, *Alistipes* and *Lactobacillus* (Figure 4D). *Lactobacillus* are a symbiotic genera of *Firmicutes*, which regulate both the adaptive and innate arms of the immune system^39^, ^40^ and have been observed to influence the severity of autoimmune disease such as EAE and allergic inflammation.^41^, ^42^, ^43^ Further analysis revealed the presence of 6 *Lactobacilli* species in the fecal samples (Figure 4E). In naïve animals the abundance of each species peaked during the rest phase (ZT4‐8). In CIA, some species maintained this peak (*L. faecis* and *L. salivarius*), but others exhibited reduced amplitude during the day (*L. johnsonii*, *L. reuteri* and *L. gasseri*) or a shift in the peak (*L. intestinalis*).

### 3.5 Arthritic mice exhibit altered profiles of microbial metabolites

**FIGURE 5:**
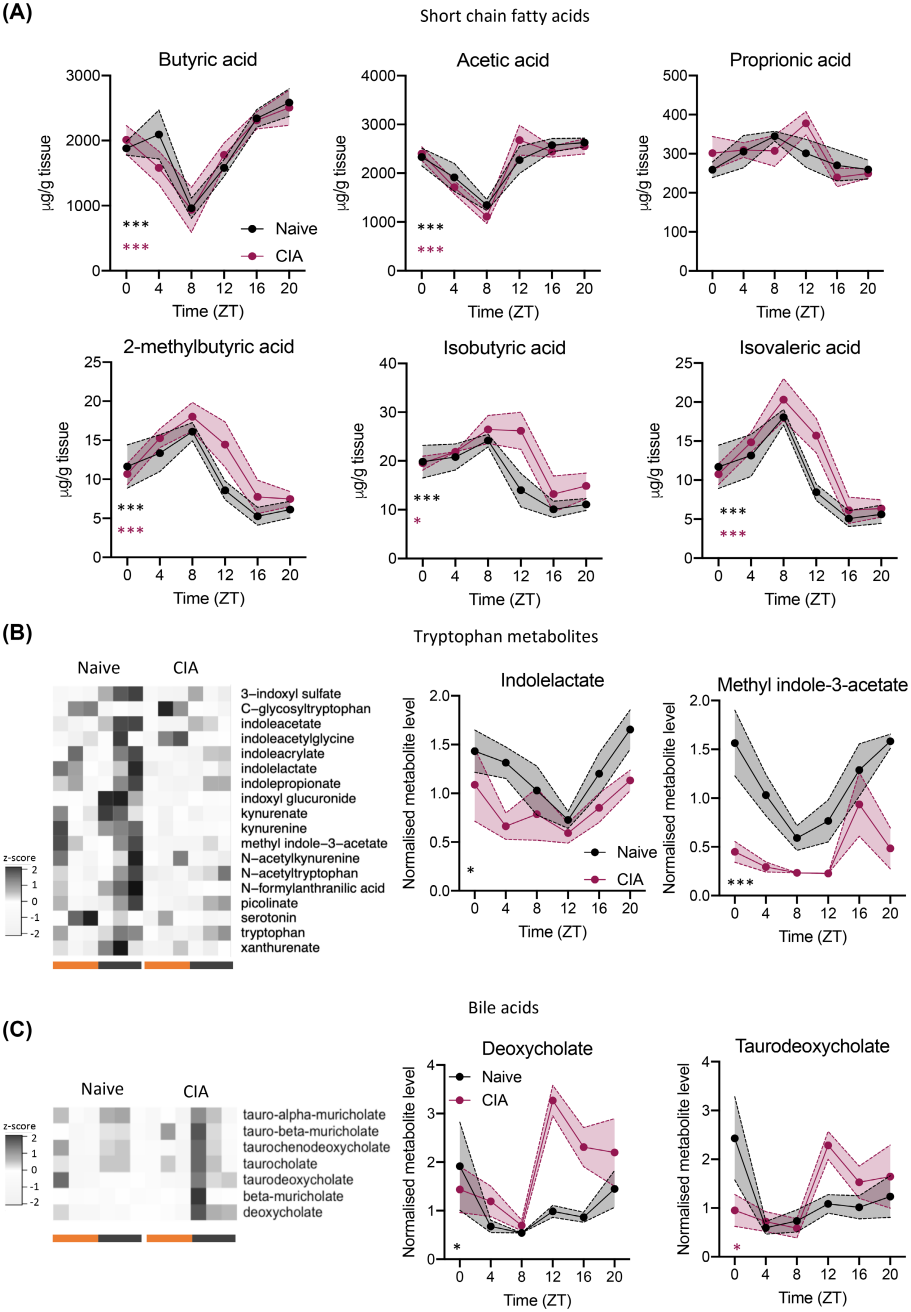
Circadian profiles of microbial metabolites are altered in inflammatory arthritis. (A) Metabolomic analysis of short chain fatty acids (SCFA) in caecal samples from naïve and CIA mice over the 24 h day, *n* = 5/time point, values are mean ± SEM, asterix denotes significant 24 h rhythms (assessed using JTK_Cycle) whereby *padj < .05, **padj < .01 and ***padj < .005. Metabolomic analysis of (B) tryptophan metabolites and (C) bile acids in plasma samples from naïve and CIA mice over the 24 h day, *n* = 5/time point, values are mean ± SEM asterix denotes significant 24 h rhythms (assessed using JTK_Cycle) whereby *padj < .05, **padj < .01 and ***padj < .005.

We next focused on the consequences of CIA on circadian variation in microbial metabolites, which are well recognized for modulating immunity.^44^ First, we focused on short chain fatty acids (SCFAs) within the caecum, a major site for bacterial fermentation of non‐digestible dietary fiber (Figure 5A and Table S3). Results revealed that 5 of the 8 SCFA analyzed exhibited circadian variation in naïve animals (JTK_Cycle *p* adj < .05). Butyric acid and acetic acid peaked at ZT20 whereas, isovaleric acid, isobutyric acid and 2‐methylbutyric acid peaked at ZT6‐8. In the setting of CIA, 24 h rhythmicity in caecal SCFAs persisted. In addition to this targeted approach, metabolomic profiling of small polar metabolites was carried out on plasma samples from naïve and arthritic mice. Here we focused on two specific classes of immune modulatory compounds—tryptophan metabolites and bile acids (BAs) (Figure 5B,C and Table S4). (The full dataset has been published elsewhere^29^). We detected that 13 of the 18 tryptophan metabolites in the plasma samples were rhythmic in naïve animals. Of these several are known to be influenced by the microbiome: 3‐indoxyl sulfate, indoleacetate, indole acrylate, indolelactate, indole proprionate, N‐acetyltryptophan, and methyl indole‐3‐acetate. These microbial metabolites were altered in CIA, with loss of rhythmicity in indolelactate and methyl indole‐3‐acetate. Of the 7 modified BAs detected in the plasma, deoxycholate and taurodeoxycholate are secondary BAs transformed by the microbiota. Intriguingly, both exhibited altered rhythmicity in CIA with higher circulating levels in the dark phase in arthritic mice. Together, these data suggest that chronic inflammatory arthritis induces fundamental changes in the rhythmicity of circulating microbial metabolites, with a striking selectivity of action.

## 4 DISCUSSION

The circadian clock directs rhythms in physiological processes within the colon. In the setting of arthritis, a number of these circadian regulated processes, critical for maintaining immune homeostasis, become disrupted. This occurs in the absence of local colonic inflammation and with minimal disruption of the core clock. This is in line with earlier observations that immune insults (such as tumorigenesis or depletion of the microbiota) can re‐programme distal circadian processes in the absence of changes to the core clock.^15^, ^45^ In addition to this temporal re‐organization, our results highlighted significant up‐regulation of genes involved in tight junctions. Together these data indicate detrimental effects on gut barrier function.

Barrier permeability was affected by arthritis. Naive animals exhibited robust daily variation in fecal albumin levels, peaking at ZT8, which complements observations in the small intestines.^19^ Daytime barrier permeability was enhanced in arthritic mice. The noted discordance between gene expression (heightened gene expression in CIA) and barrier function (leaky barrier) may indicate compensatory up‐regulation of tight‐junction associated genes in a partially successful attempt to maintain barrier function. These observations in mouse CIA correlate with recent data from RA patients showing increased levels of markers for gut‐permeability in the serum, including bacterial products, compared to healthy controls.^46^, ^47^ Our data highlights the temporally dynamic nature of colonic permeability, revealing that whilst arthritis affects barrier permeability, rhythms are maintained. Others have reported early changes in gut permeability in CIA induced by zonulin, which is produced by IECs and promotes disengagement of ZO‐1 protein from the tight junction complex.^47^ We were unable to reliably detect zonulin in blood samples from our animals to examine this potential mechanism in our experimental cohorts. Increased barrier permeability during disease initiation is reported to be accompanied by increased frequencies of Th1 and Th17 cells in the small intestines, which have the potential to migrate to the joints.^47^ Our studies here did not reveal any significant changes in total CD4^+^ T cell numbers within the colonic lamina propria of mice with established arthritis, suggesting maintained barrier leakiness in established disease in the absence of intestinal inflammation. However, whilst numbers do not change, we cannot rule out altered CD4+ T cell function. We also note that here we restricted our analysis to examining cellular changes within the colonic lamina propria. Other studies in mouse models of arthritis have detected the presence of inflammatory cell infiltrates in the small intestines.^46^, ^47^

Bacteria 16S rRNA sequencing revealed temporal re‐organization of the gut microbiota in arthritis. We did not see any significant difference in the diversity of this community, instead genus specific dampening of daily oscillations in abundance. Heddes and colleagues recently revealed a critical role for the IEC clock in driving 24 h rhythms in a significant proportion of the microbiota and its metabolic outputs.^22^ Here, the temporal re‐organization of the microbiota occurs in the presence of a robust gut clock, thus we do not see this as a consequence of intestinal clock dampening. We observed loss of 24 h rhythmicity within *Alistipes* and *Odoribacter* (both *Bacteroidetes*) and *Lactobacillus* (a *Firmicute*). Whilst other murine and human studies have taken a “snapshot” approach to assess microbial changes in the setting or inflammatory arthritis, here we take in a broader perspective assessing abundance around the clock. Given the circadian dynamics of this community and our growing understanding of the importance of these oscillations to host health^15^, ^17^, ^20^ this approach is necessary to fully address host‐microbiome interactions and their contribution to development and perpetuation of inflammatory arthritis, and potentially co‐morbidities. Taking into account the high amplitude oscillations in a significant proportion of the gut microbiota, without knowing the time of day at which the community was sampled in previous studies, it is hard to draw comparisons between previous work and data presented here. Of note however, a previous study in RA patients has identified reduced abundance of *Odoribacter* and *Lactobacillus* compared to healthy controls.^48^ Recent work has linked arrhythmic taxa as a predictor of Type 2 diabetes^20^ and our data here further supports the notion that loss of rhythmicity in the microbiota is associated with disease. Loss of rhythms within the microbiota in the setting of CIA may impact disease progression and the development of co‐morbidities. As an example, we know that loss in diurnal oscillations of the microbiota alters host metabolic homeostasis.^21^

We further focused on investigating changes in rhythms within *Lactobacilli* in the setting of arthritis. These *Firmicutes* have been linked to the development of arthritis^49^ and dietary supplementation with certain species has a protective effect in animal models of arthritis.^50^, ^51^ Our data demonstrates dampening of the peak abundance (ZT8) of species of *Lactobacillus*, including *L. reuteri*. *L. reuteri* can enhance intestinal barrier function^52^; promote dendritic cell maturation and IL10 production^53^; has protective effects in allergic lung inflammation^42^ and colitis^54^; and conversely has recently been linked to exacerbations of EAE.^43^ With such widespread functions, it will be pertinent in the future to understand the consequence of loss of rhythms in this species on barrier function and immune cell populations.

Whilst it is clear that microbes within the gut can themselves induce changes to host immunity, microbial metabolites are emerging to play a critical role. This includes SCFA, tryptophan metabolites and secondary bile acids.^12^ There is growing recognition that SCFA concentrations within biological samples exhibit 24 h variation.^55^, ^56^, ^57^ We show here 24 h rhythmicity across multiple classes of microbial metabolites, and for the first time, the consequence of inflammatory arthritis on this rhythmicity. SCFAs are derived from bacterial anaerobic fermentation of dietary fiber, and have a plethora of immunomodulatory functions impacting on a vast range of cell types.^58^ We identified robust rhythmicity in caecal SCFA concentrations, which persists in CIA. Within the plasma, we observed loss of rhythmicity of two tryptophan metabolites with established anti‐inflammatory activity: indolelactate^59^ and methyl indole‐3‐acetate (a methyl ester of indole‐3‐acetic acid which itself has been shown to promote IL‐22 production and barrier maintenance^60^ and has protective effects in a mouse model of ankylosing spondylitis^61^). Tryptophan metabolites are produced by mouse and human gut bacteria including *L. reuteri* and *Clostridium sporogenes* ^39^, ^62^, ^63^ thus it is feasible that the loss of rhythmicity in *L. reuteri* contributes to dampened rhythms in these indoles. Analysis of microbial metabolites from a global metabolite screen also identified changes in circulating levels of two secondary bile acids, deoxycholate and taurodeoxycholate. Secondary bile acids are implicated in the regulation of T cell subsets within the lamina propria, colon and periphery^64^, ^65^, ^66^ and more specifically high levels of deoxycholate has been linked to intestinal barrier dysfunction.^67^ Taken together these data implicate temporal remodeling not only in the microbial composition of the gut but also metabolic outputs. It is established that rhythmicity within the gut microbiome is important for metabolic health and we postulate that the same may be true for immune health.

These studies reveal that temporal co‐ordination of the gut transcriptome is re‐modeled in a mouse model of chronic inflammatory arthritis in the absence of local inflammation and with minimal changes to the colonic core molecular clock. Animals with established disease exhibit enhanced gut barrier permeability and loss of rhythms within bacterial taxa and microbial metabolites with known benefits to the host. Determining underlying causality within the CIA associated disturbances in gut permeability, microbiome rhythmicity and colon transcriptome is difficult to assess given the interdependent relationship between host gut function and its microbiota. There is also evidence to suggest that changes in barrier function within the colon may occur during the initial development of CIA, and persist during the chronic stages of disease. During early disease establishment significant shifts occur in the composition and diversity of the intestinal microbiota and its metabolic outputs^46^, ^47^, ^68^, ^69^ which are recognized to mediate increased intestinal permeability.^47^ Persistent joint inflammation and the associated elevation and rhythmicity in circulating cytokines including IL6, IL1β and interferon γ (IFNγ)^29^, ^30^ are likely to perpetuate altered gut function. IFNγ can downregulate IL10 receptor expression on IECs, which promotes reduced gut permeability.^46^ Similarly, IL6 and IL1β are associated with modulation of tight junction permeability at the intestinal epithelial barrier.^70^, ^71^ We postulate that changes in barrier permeability precede altered circadian regulation of the microbiome, which in turn feeds back onto the gut transcriptome. Certainly a loss of rhythms in the microbiota can lead to altered circadian expression of genes and proteins within the gut.^15^, ^17^ Further work is warranted to assess mechanisms underlying cross‐talk between the inflamed joint, the colon and its constituents, to address what is driving altered circadian regulation of the gut microbiome in arthritis.

It is now established that rhythms in the microbiome are important for optimal metabolism. Given the influence of the microbiome over the development and maintenance of the immune system, we postulate that a rhythmic microbiome is equally important for immunity, and disruption of these rhythms may negatively impact development, progression and resolution of arthritis and even disease treatment.

## AUTHOR CONTRIBUTIONS

Julie Elizabeth Gibbs, David A. Bechtold and Matthew R. Hepworth conceived and designed the research. Devin Amanda Simpkins, Polly Downton, Suzanna H. Dickson, Kathryn J. Gray and Julie Elizabeth Gibbs performed the research and acquired the data. Devin Amanda Simpkins, Robert J. Maidstone and Julie Elizabeth Gibbs analyzed and interpreted the data. Devin Amanda Simpkins, Polly Downton, David W. Ray, Joanne E. Konkel, Matthew R. Hepworth, David A. Bechtold and Julie Elizabeth Gibbs drafted and revised the manuscript.

## FUNDING INFORMATION

This work has also been funded through grants from the MRC (MR/S002715/1 to JEG, DWR and JEK; and MR/P023576/1 to DWR, DAB and JEG). JEG is a Versus Arthritis Senior Fellow (22625). MRH is supported by a Sir Henry Dale Fellowship jointly funded by the Wellcome Trust and the Royal Society (105644/Z/14/Z), BBSRC (BB/T014482/1) and Lister Institute of Preventable Medicine Prize. JEK is also funded by the BBSRC (BB/M025977/1), Lister Institute of Preventable Medicine and Versus Arthritis. DWR is also funded by the Wellcome Trust (107849/Z/15/Z).

## DISCLOSURES

The authors declare no conflicts of interest.

## Supporting information


Table S1.

Figure S1.

## Data Availability

The mouse colon RNASeq data that support the findings of this study will be openly available from 15/03/2023 at www.ncbi.nlm.nih.gove/geo record GSE184233 (Consequences of collagen induced inflammatory arthritis on circadian regulation of the gut microbiome). The 16S rRNA data is available upon reasonable request.
