# Supplementary material for: Consequences of collagen induced inflammatory arthritis on circadian regulation of the gut microbiome
Source: FASEB J. 2022 Dec 15;37(1):e22704. doi: 10.1096/fj.202201728R (PMC10107696; doi:10.1096/fj.202201728R)
Supplement: Supplementary file 1 — Table S1. [file FSB2-37-e22704-s001.docx]

**Supplementary Table 1:** Packages used in R Studio analysis

| **Method** | **Package name** | **Ref** | **URL** |
| --- | --- | --- | --- |
| RNA Sequencing  / 16s analysis | Bioformat | [1] | https://github.com/joey711/biomformat/ |
|  |  |  | http://biom-format.org/. |
| RNA Sequencing | RColorBrewer | [2] | https://CRAN.R-project.org/package=RColorBrewer |
| RNA Sequencing | compareRhythms | [3] | https://github.com/bharathananth/compareRhythms |
| RNA Sequencing | clusterProfiler | [4] | https://www.bioconductor.org/packages/clusterProfiler |
| RNA Sequencing | limma | [5] | https://git.bioconductor.org/packages/limma |
| RNA Sequencing | dplyr | [6] | https://CRAN.R-project.org/package=dplyr |
| RNA Sequencing | tidyverse | [7] | <https://doi.org/10.21105/joss.01686> |
| RNA Sequencing | MAGeCKFlute | [8] | https://git.bioconductor.org/packages/MAGeCKFlute |
| RNA Sequencing | pathview | [9] | http: //bioinformatics.oxfordjournals.org/content/29/14/1830.full. |
| RNA Sequencing | ggplot2 | [10] | http://ggplot2.tidyverse.org, https://github.com/tidyverse/ggplot2 |
| RNA Sequencing / 16s analysis | gplots | [11] | https://CRAN.R-project.org/package=gplots |
| RNA Sequencing | patchwork | [12] | https://CRAN.R-project.org/package=patchwork |
| RNA Sequencing | ggforce | [13] | https://CRAN.R-project.org/package=ggforce |
| 16s analysis | tibble | [14] | https://CRAN.R-project.org/package=tibble |
| 16s analysis | reshape | [15] | http://www.jstatsoft.org/v21/i12/paper |
| 16s analysis | stringr | [16] | https://CRAN.R-project.org/package=stringr |
| 16s analysis | plyr | [17] | http://www.jstatsoft.org/v40/i01/. |

**[1]** Paul J. McMurdie and Joseph N Paulson (2019). biomformat: An interface package for the BIOM file format. **[2]** Erich Neuwirth (2014). RColorBrewer: ColorBrewer Palettes. R package version 1.1-2. **[3]** Anne Pelikan, Hanspeter Herzel, Achim Kramer, Bharath Ananthasubramaniam bioRxiv 2020.12.18.423465. **[4]** G Yu*, LG Wang, Y Han, QY He. clusterProfiler: an R package for comparing biological themes among gene clusters.*OMICS: A Journal of Integrative Biology*2012, 16(5):284-287.***[5]** Ritchie, M.E., Phipson, B., Wu, D., Hu, Y., Law, C.W., Shi, W., and Smyth, G.K. (2015). limma powers differential expression analyses for RNA-sequencing and microarray studies. Nucleic Acids Research 43(7), e47. **[6]** Hadley Wickham, Romain François, Lionel Henry and Kirill Müller (2020). dplyr: A Grammar of Data Manipulation. R package version 1.0.0. **[7]** Wickham et al., (2019). Welcome to the tidyverse. Journal of Open Source Software, 4(43), 1686. **[8]** Binbin Wang, Mei Wang, Wubing Zhang. “Integrative analysis of pooled CRISPR genetic screens using MAGeCKFlute.” Nature Protocols (2019), doi: 10.1038/s41596-018-0113-7. R package version 1.6.5. **[9]** Weijun Luo and Cory Brouwer. Pathview: an R/Bioconductor package for pathway-based data integration and visualization. Bioinformatics, 29(14):1830-1831, 2013. **[10]** H. Wickham. ggplot2: Elegant Graphics for Data Analysis. Springer-Verlag New York, 2016. **[11]** Gregory R. Warnes, Ben Bolker, Lodewijk Bonebakker, Robert Gentleman, Wolfgang Huber, Andy Liaw, Thomas Lumley, Martin Maechler, Arni Magnusson, Steffen Moeller, Marc Schwartz and Bill Venables (2020). gplots: Various R Programming Tools for Plotting Data. R package version 3.1.0. **[12]** Thomas Lin Pedersen (2020). patchwork: The Composer of Plots. R package version 1.0.1. **[13]** Thomas Lin Pedersen (2020). ggforce: Accelerating 'ggplot2'. R package version 0.3.2. **[14]** Kirill Müller and Hadley Wickham (2020). tibble: Simple Data Frames. R package version 3.0.3. **[15]** H. Wickham. Reshaping data with the reshape package. Journal of Statistical Software, 21(12), 2007. **[16]** Hadley Wickham (2019). stringr: Simple, Consistent Wrappers for Common String Operations. R package version 1.4.0. **[17]** Hadley Wickham (2011). The Split-Apply-Combine Strategy for Data Analysis. Journal of Statistical Software, 40(1), 1-29.

**Supplementary Table 2:** Antibodies used for staining of lamina propria cells for flow cytometry.

| **Antibody** | **Clone** | **Fluorophore** | **Supplier** |
| --- | --- | --- | --- |
| CD45 | 30-F11 | BV650, BV510 | BioLegend |
| CD3e | 145-2C11 | PerCP-Cyanine5.5 | eBioscience |
| Ly6G | 1A8 | BV42, AF700 | BioLegend |
| CD64 | X54-5/7.1 | APC | BioLegend |
| CD11b | M1/70 | BV605, PE | BioLegend |
| Siglec F | E50-2440 | PE-CF594 | BD Horizon |
| MHC II | M5/114.15.2 | FITC | Biolegend |
| CD19 | 6D5 | BV650 | BioLegend |
| CD4 | RM4-5 | AF700 | BioLegend |
| CD8 | 53-6.7 | BV785 | BioLegend |
| Ly6C | al-21 | PE-Cy7 | BD Pharmigen |

**Supplementary Table 3:** JTK_Cycle analysis of Caecal concentrations of SCFA

|  | **Naive** | | **CIA** | |
| --- | --- | --- | --- | --- |
| **SCFA** | ***Adj P*** | ***Lag*** | ***Adj P*** | ***Lag*** |
| Acetic acid | 0.0011 | 20 | 0.000070 | 20 |
| Proprionic acid | 0.20 | 10 | 0.18 | 10 |
| Butyric acid | 0.000085 | 20 | 0.00020 | 20 |
| Isovaleric acid | 0.00015 | 8 | 0.000002 | 8 |
| Isobutyric acid | 0.0013 | 6 | 0.013 | 8 |
| 2-Methylbutyric acid | 0.000057 | 8 | 0.00011 | 8 |
| Valeric acid | 0.80 | 18 | 0.47 | 10 |
| Hexanoic acid | 1.000000 | 0 | 0.30 | 22 |

|  | **Naïve** | | **CIA** | |
| --- | --- | --- | --- | --- |
| **Tryptophan metabolite** | ***Adj P*** | ***Lag*** | ***Adj P*** | ***Lag*** |
| 3-indoxyl sulfate* | 0.000051 | 18 | 0.021 | 18 |
| C-glycosyltryptophan | 0.14 | 8 | 0.000094 | 8 |
| Indoleacetate* | 0.0015 | 18 | 0.046 | 18 |
| Indoleacetylglycine | 0.039 | 18 | 1.00 | 18 |
| Indoleacrylate* | 0.031 | 20 | 0.018 | 22 |
| Indolelactate* | 0.020 | 0 | 0.089 | 22 |
| Indolepropionate* | 0.0019 | 22 | 0.0000055 | 20 |
| Indoxyl glucuronide | 0.0073 | 18 | 0.26 | 18 |
| Kynurenate | 0.72 | 16 | 0.95 | 20 |
| Kynurenine | 0.14 | 20 | 0.35 | 18 |
| Methyl indole-3-acetate* | 0.00022 | 22 | 0.67 | 22 |
| N-acetylkynurenine | 0.026 | 20 | 1.0 | 6 |
| N-acetyltryptophan* | 0.0000055 | 20 | 0.019 | 20 |
| N-formylanthranilic acid | 0.00042 | 20 | 0.00039 | 18 |
| Picolinate | 0.00093 | 20 | 0.0059 | 18 |
| Serotonin* | 1.00 | 6 | 0.86 | 10 |
| Tryptophan | 0.000041 | 20 | 0.000034 | 18 |
| Xanthurenate | 0.068 | 18 | 0.061 | 18 |
| **Bile acids** | ***Adj P*** | ***Lag*** | ***Adj P*** | ***Lag*** |
| Tauro-alpha muricholate | 1.00 | 18 | 0.072 | 14 |
| Tauro-beta-muricholate | 0.28 | 18 | 0.0028 | 16 |
| Taurochenodeoxycholate | 0.30 | 16 | 0.10 | 16 |
| Taurocholate | 0.25 | 18 | 0.10 | 16 |
| Taurodeoxycholate* | 1.00 | 22 | 0.027 | 16 |
| Beta muricholate | 1.00 | 22 | 0.080 | 14 |
| Deoxycholate* | 0.014 | 20 | 0.13 | 16 |

**Supplementary Table 4:** JTK_Cycle analysis of serum concentrations of microbial metabolites

*Associated with the microbiota
