# Supplementary material for: Consequences of collagen induced inflammatory arthritis on circadian regulation of the gut microbiome
Source: FASEB J. 2022 Dec 15;37(1):e22704. doi: 10.1096/fj.202201728R (PMC10107696; doi:10.1096/fj.202201728R)
Supplement: Supplementary file 2 — Figure S1. [file FSB2-37-e22704-s002.pdf]

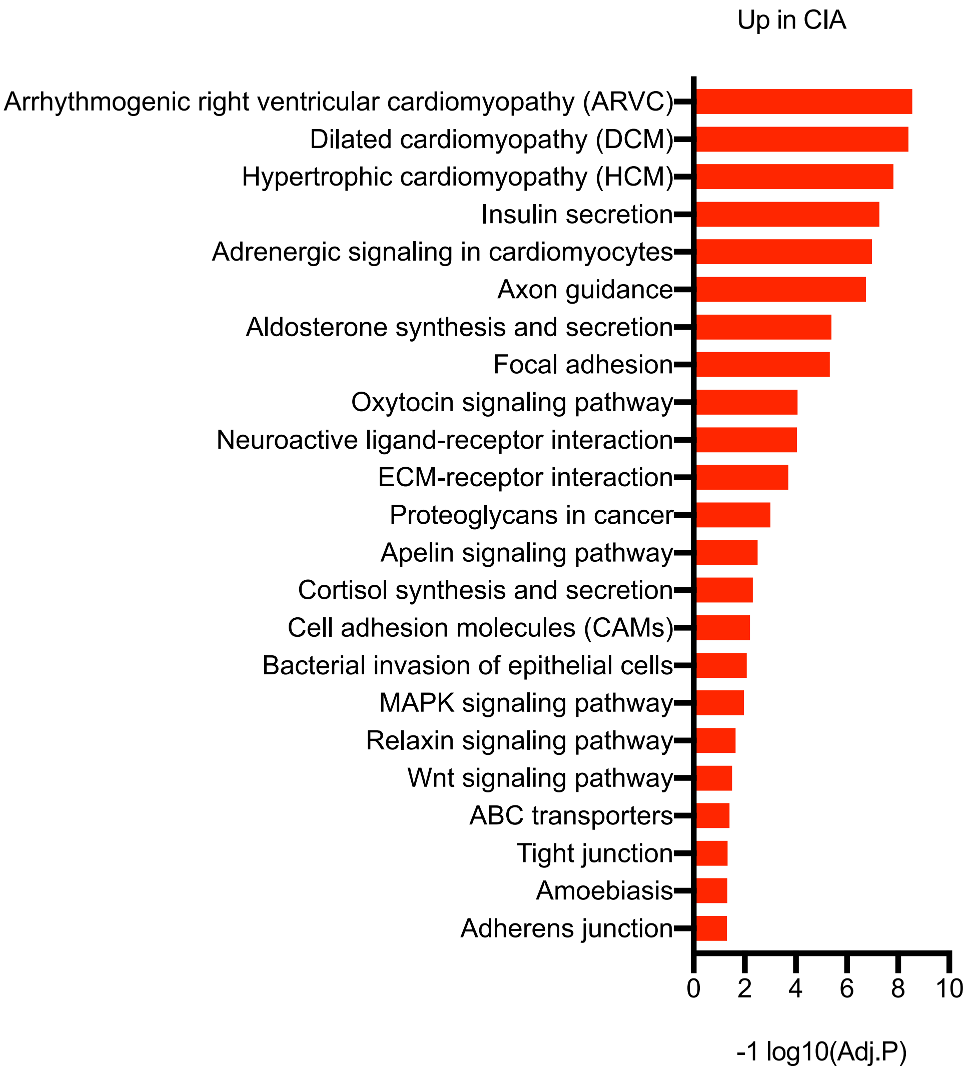

Supplementary Figure 1: Pathway analysis (KEGG mouse 2019, Padj<0.05) of transcripts that are up in colonic tissue from CIA mice compared to naïve tissue

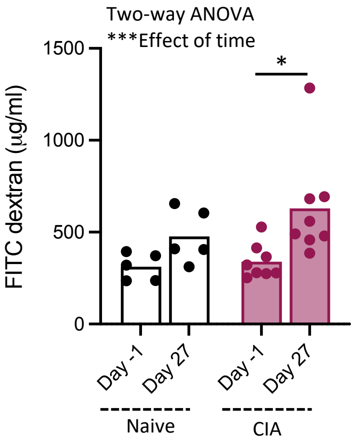

Supplementary Figure 2: FITC dextran concentrations in serum post oral gavage at ZT0 in naïve mice (n=5) and CIA mice (n=8) prior to collagen immunisation (day -1, CIA only) and after development of symptoms in CIA mice (day 27 onwards). Two-way ANOVA (effect of time P=0.005) with post-hoc Tukey multiple comparison tests.

Supplementary Figure 3

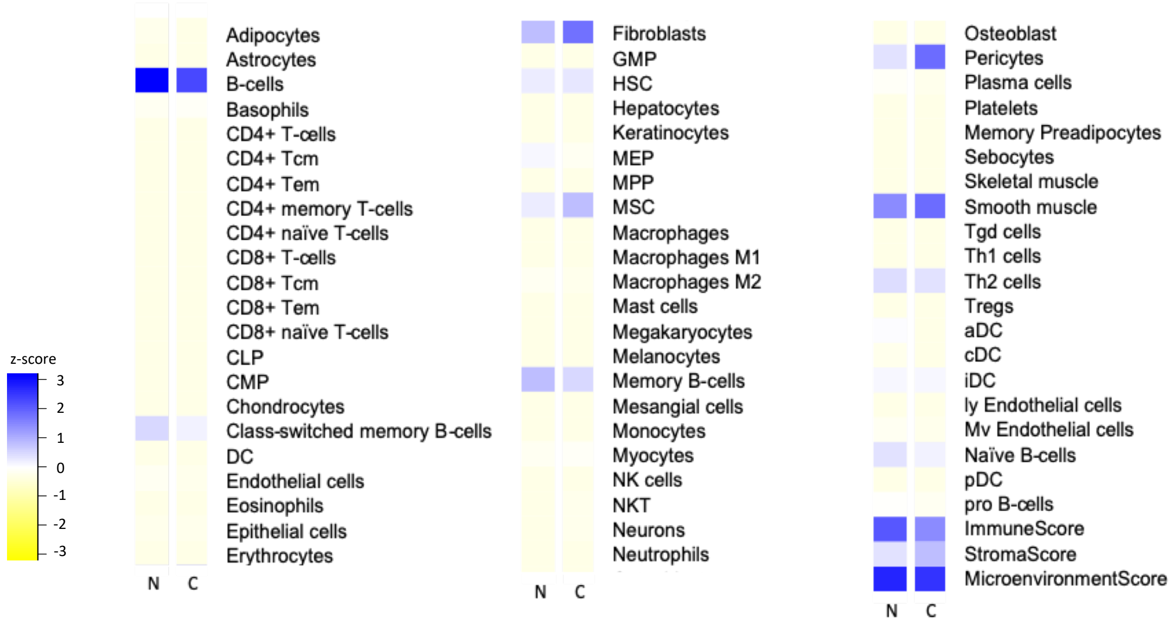

Supplementary Figure 3: X cell enrichment analysis from gut RNAseq data was used to associate gene expression to related cell types. Data is averaged from 5 replicates and 6 time points.

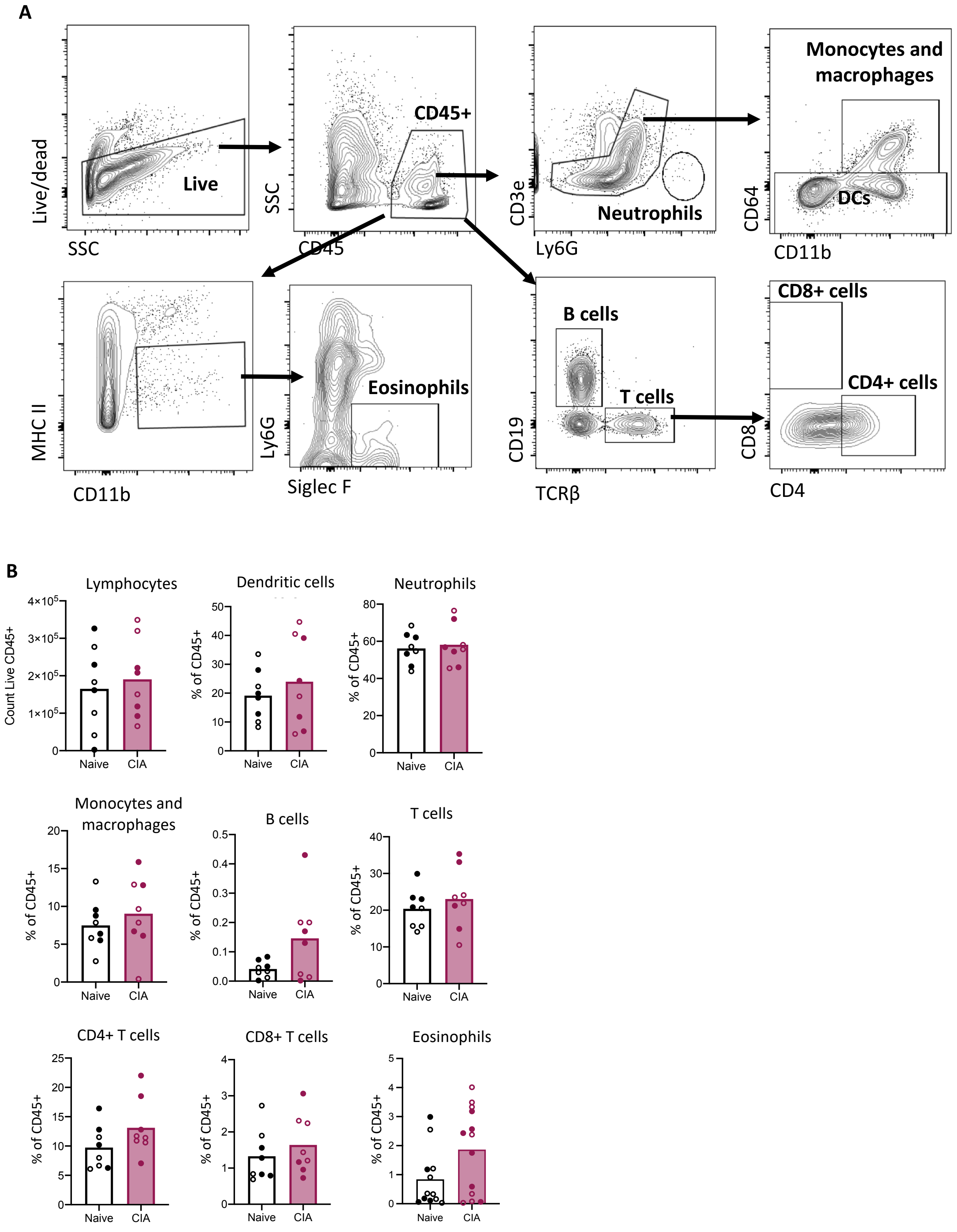

Supplementary Figure 4: (A) Gating strategy for identification of immune cell populations within the colonic lamina propria. All gates were informed by fluorescence-minus one (FMO) controls. (B) Quantification of immune cell populations within the colonic lamina propria in naïve (black) and arthritic (maroon) animals at ZT 8 (open circles) and ZT20 (closed circles) n=8/treatment in all except eosinophils where naive; n=12 and CIA; n=13.

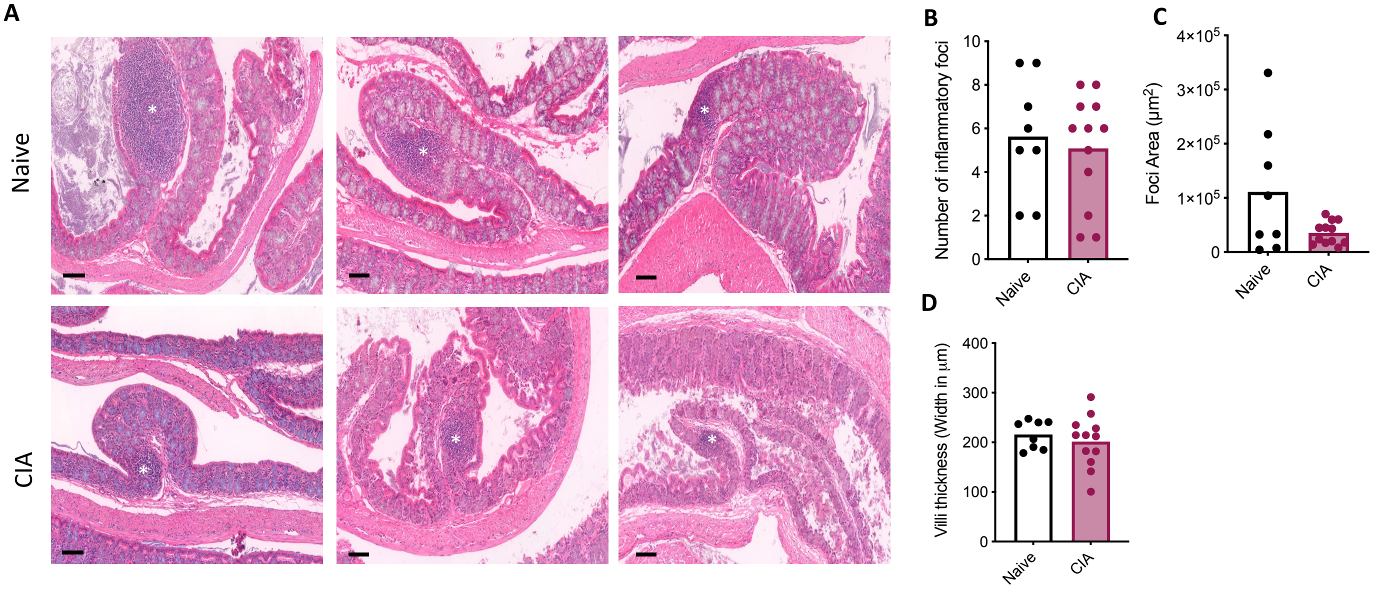

Supplementary Figure 5: (A) Histological analysis of lamina propria. Representative histology images of samples stained with H and E. Scale represents 100 µm. * shows representative foci of inflammation. (B) Total number of inflammatory foci per slide, naive (n=8), CIA (n=12). (C) Area of each inflammatory foci, each point is the average area of all foci in each sample, naive (n=8), CIA (n=12). (D) Villi thickness, each point is the average of 3 regions of interest, naive (n=8) and CIA (n=12).

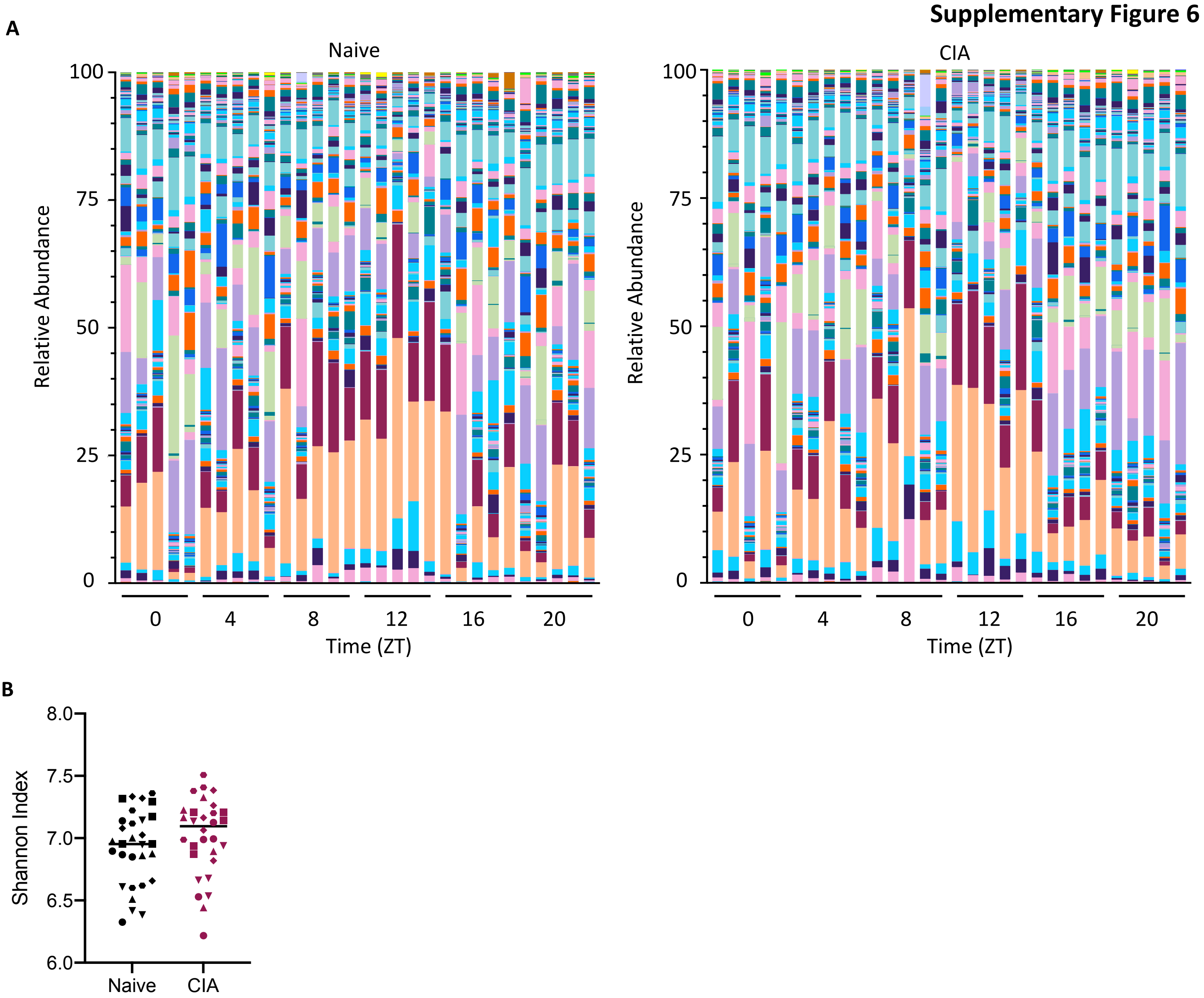

Supplementary Figure 6: (A) Relative abundance at the species level as determined by 16S rRNA sequencing in faecal samples collected from naïve and CIA mice across the circadian day (n=5 mice per time point). Each colour represents a different species (see Supplemental Figure 8 for full legend). (B) Comparison of species diversity (Shannon index) between samples from naïve and CIA mice (n=30 per group). Circle =ZT0, square=ZT4, triangle=ZT8, downward triangle=ZT12, hexagon=ZT16 and diamond=ZT20.

Supplementary Figure 7

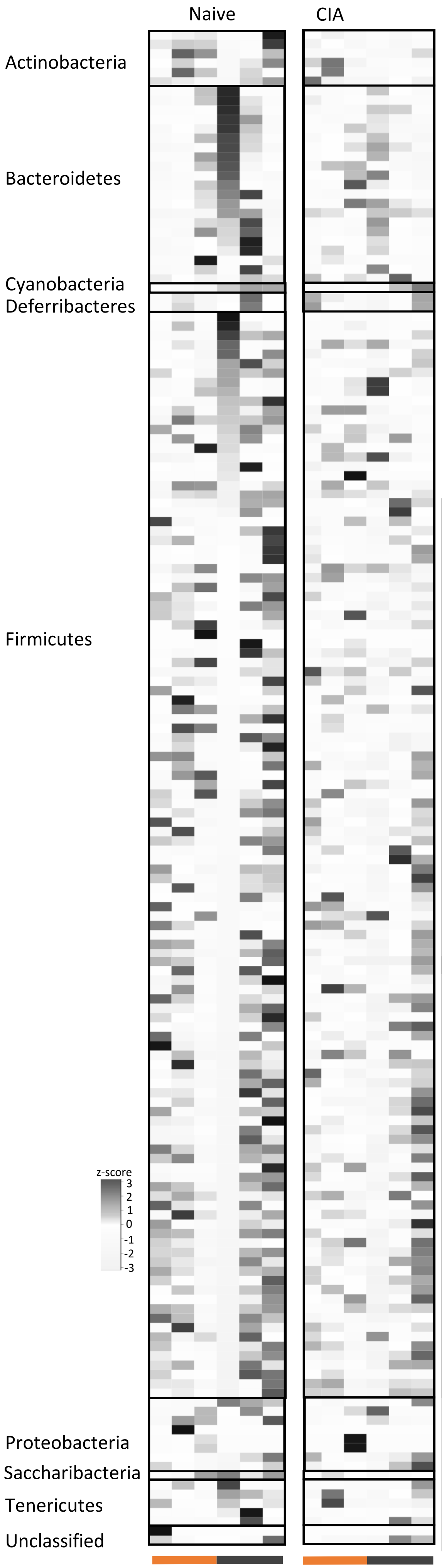

Supplementary Figure 7: Heat map illustrating the temporal organization of microbial species (organized by phylum) within the gut microbiota across the 24h day (orange: ZT0, ZT4 and ZT8 and black: ZT12, ZT16, ZT20), n=5/time point.

Legend for relative abundance at species level (Supplementary Figure 6) Naïve samples

k_Bacteria;p_Actinobacteria;c_Coriobacteriia;o_Coriobacteriales;f_Coriobacteriaceae;g_Enterorhabdus;s_Enterorhabdus mucosicola
k_Bacteria;p_Actinobacteria;c_Coriobacteriia;o_Coriobacteriales;f_Coriobacteriaceae;g_Enterorhabdus;s_mouse gut metagenome
k_Bacteria;p_Actinobacteria;c_Coriobacteriia;o_Coriobacteriales;f_Coriobacteriaceae;g_Enterorhabdus;s_uncultured bacterium
k_Bacteria;p_Actinobacteria;c_Coriobacteriia;o_Coriobacteriales;f_Coriobacteriaceae;g_Enterorhabdus;s_unidentified
k_Bacteria;p_Actinobacteria;c_Coriobacteriia;o_Coriobacteriales;f_Coriobacteriaceae;g_uncultured;s_uncultured Coriobacteriales bacterium
k_Bacteria;p_Actinobacteria;c_Coriobacteriia;o_Coriobacteriales;f_Coriobacteriaceae;NA;NA
k_Bacteria;p_Bacteroidetes;c_Bacteroidia;o_Bacteroidales;f_Bacteroidaceae;g_Bacteroides;NA
k_Bacteria;p_Bacteroidetes;c_Bacteroidia;o_Bacteroidales;f_Bacteroidaceae;g_Bacteroides;s_Bacteroides acidifaciens
k_Bacteria;p_Bacteroidetes;c_Bacteroidia;o_Bacteroidales;f_Bacteroidaceae;g_Bacteroides;s_human gut metagenome
k_Bacteria;p_Bacteroidetes;c_Bacteroidia;o_Bacteroidales;f_Bacteroidaceae;g_Bacteroides;s_unidentified
k_Bacteria;p_Bacteroidetes;c_Bacteroidia;o_Bacteroidales;f_Bacteroidales S24-7 group;g_mouse gut metagenome;s_mouse gut metagenome
k_Bacteria;p_Bacteroidetes;c_Bacteroidia;o_Bacteroidales;f_Bacteroidales S24-7 group;g_uncultured bacterium;s_uncultured bacterium
k_Bacteria;p_Bacteroidetes;c_Bacteroidia;o_Bacteroidales;f_Bacteroidales S24-7 group;g_uncultured Bacteroidales bacterium;s_uncultured Bacteroidales bacterium
k_Bacteria;p_Bacteroidetes;c_Bacteroidia;o_Bacteroidales;f_Bacteroidales S24-7 group;g_uncultured organism;s_uncultured organism
k_Bacteria;p_Bacteroidetes;c_Bacteroidia;o_Bacteroidales;f_Bacteroidales S24-7 group;NA;NA
k_Bacteria;p_Bacteroidetes;c_Bacteroidia;o_Bacteroidales;f_Porphyromonadaceae;g_Odoribacter;s_uncultured bacterium
k_Bacteria;p_Bacteroidetes;c_Bacteroidia;o_Bacteroidales;f_Porphyromonadaceae;g_Odoribacter;s_unidentified
k_Bacteria;p_Bacteroidetes;c_Bacteroidia;o_Bacteroidales;f_Porphyromonadaceae;g_Parabacteroides;NA
k_Bacteria;p_Bacteroidetes;c_Bacteroidia;o_Bacteroidales;f_Porphyromonadaceae;g_Parabacteroides;s_Parabacteroides goldsteinii
k_Bacteria;p_Bacteroidetes;c_Bacteroidia;o_Bacteroidales;f_Prevotellaceae;g_Prevotellaceae UCG-001;s_uncultured bacterium
k_Bacteria;p_Bacteroidetes;c_Bacteroidia;o_Bacteroidales;f_Rikenellaceae;g_Alistipes;NA
k_Bacteria;p_Bacteroidetes;c_Bacteroidia;o_Bacteroidales;f_Rikenellaceae;g_Alistipes;s_Bacteroidales bacterium ph8
k_Bacteria;p_Bacteroidetes;c_Bacteroidia;o_Bacteroidales;f_Rikenellaceae;g_Alistipes;s_uncultured bacterium
k_Bacteria;p_Bacteroidetes;c_Bacteroidia;o_Bacteroidales;f_Rikenellaceae;g_Alistipes;s_uncultured organism
k_Bacteria;p_Bacteroidetes;c_Bacteroidia;o_Bacteroidales;f_Rikenellaceae;g_Alistipes;s_unidentified
k_Bacteria;p_Bacteroidetes;c_Bacteroidia;o_Bacteroidales;f_Rikenellaceae;g_Rikenella;s_uncultured bacterium
k_Bacteria;p_Bacteroidetes;c_Bacteroidia;o_Bacteroidales;f_Rikenellaceae;g_Rikenellaceae RC9 gut group;s_uncultured bacterium
k_Bacteria;p_Cyanobacteria;c_Chloroplast;o_Phaseolus acutifolius (tepary bean);f_Phaseolus acutifolius (tepary bean);g_Phaseolus acutifolius (tepary bean);s_Phaseolus acutifolius (tepary bean)
k_Bacteria;p_Deferribacteres;c_Deferribacteres;o_Deferribacterales;f_Deferribacteraceae;g_Mucispirillum;NA
k_Bacteria;p_Deferribacteres;c_Deferribacteres;o_Deferribacterales;f_Deferribacteraceae;g_Mucispirillum;s_uncultured bacterium
k_Bacteria;p_Firmicutes;c_Bacilli;o_Lactobacillales;f_Enterococcaceae;g_Enterococcus;NA
k_Bacteria;p_Firmicutes;c_Bacilli;o_Lactobacillales;f_Lactobacillaceae;g_Lactobacillus;s_Lactobacillus faecis
k_Bacteria;p_Firmicutes;c_Bacilli;o_Lactobacillales;f_Lactobacillaceae;g_Lactobacillus;s_Lactobacillus gasseri
k_Bacteria;p_Firmicutes;c_Bacilli;o_Lactobacillales;f_Lactobacillaceae;g_Lactobacillus;s_Lactobacillus intestinalis
k_Bacteria;p_Firmicutes;c_Bacilli;o_Lactobacillales;f_Lactobacillaceae;g_Lactobacillus;s_Lactobacillus johnsonii
k_Bacteria;p_Firmicutes;c_Bacilli;o_Lactobacillales;f_Lactobacillaceae;g_Lactobacillus;s_Lactobacillus reuteri
k_Bacteria;p_Firmicutes;c_Bacilli;o_Lactobacillales;f_Lactobacillaceae;g_Lactobacillus;s_Lactobacillus salivarius
k_Bacteria;p_Firmicutes;c_Bacilli;o_Lactobacillales;f_Streptococcaceae;g_Streptococcus;s_Streptococcus danieliae
k_Bacteria;p_Firmicutes;c_Clostridia;o_Clostridiales;f_Clostridiales vadinBB60 group;g_Clostridiales bacterium JN18_A56_K;s_Clostridiales bacterium JN18_A56_K
k_Bacteria;p_Firmicutes;c_Clostridia;o_Clostridiales;f_Clostridiales vadinBB60 group;g_uncultured bacterium;s_uncultured bacterium
k_Bacteria;p_Firmicutes;c_Clostridia;o_Clostridiales;f_Clostridiales vadinBB60 group;g_unidentified;s_unidentified
k_Bacteria;p_Firmicutes;c_Clostridia;o_Clostridiales;f_Clostridiales vadinBB60 group;NA;NA
k_Bacteria;p_Firmicutes;c_Clostridia;o_Clostridiales;f_Family XIII;g_[Eubacterium] brachy group;s_uncultured bacterium
k_Bacteria;p_Firmicutes;c_Clostridia;o_Clostridiales;f_Family XIII;g_[Eubacterium] nodatum group;s_uncultured organism
k_Bacteria;p_Firmicutes;c_Clostridia;o_Clostridiales;f_Family XIII;g_Family XIII AD3011 group;s_uncultured bacterium
k_Bacteria;p_Firmicutes;c_Clostridia;o_Clostridiales;f_Family XIII;g_Family XIII UCG-001;s_uncultured bacterium
k_Bacteria;p_Firmicutes;c_Clostridia;o_Clostridiales;f_Lachnospiraceae;g_Acetatifactor;NA
k_Bacteria;p_Firmicutes;c_Clostridia;o_Clostridiales;f_Lachnospiraceae;g_Acetatifactor;s_uncultured bacterium
k_Bacteria;p_Firmicutes;c_Clostridia;o_Clostridiales;f_Lachnospiraceae;g_Blautia;NA
k_Bacteria;p_Firmicutes;c_Clostridia;o_Clostridiales;f_Lachnospiraceae;g_Blautia;s_Lachnospiraceae bacterium 615
k_Bacteria;p_Firmicutes;c_Clostridia;o_Clostridiales;f_Lachnospiraceae;g_Coprococcus 1;s_uncultured bacterium
k_Bacteria;p_Firmicutes;c_Clostridia;o_Clostridiales;f_Lachnospiraceae;g_Coprococcus 1;s_unidentified
k_Bacteria;p_Firmicutes;c_Clostridia;o_Clostridiales;f_Lachnospiraceae;g_Incertae Sedis;s_uncultured bacterium
k_Bacteria;p_Firmicutes;c_Clostridia;o_Clostridiales;f_Lachnospiraceae;g_Lachnoclostridium;NA
k_Bacteria;p_Firmicutes;c_Clostridia;o_Clostridiales;f_Lachnospiraceae;g_Lachnoclostridium;s_[Clostridium] scindens
k_Bacteria;p_Firmicutes;c_Clostridia;o_Clostridiales;f_Lachnospiraceae;g_Lachnoclostridium;s_uncultured bacterium
k_Bacteria;p_Firmicutes;c_Clostridia;o_Clostridiales;f_Lachnospiraceae;g_Lachnoclostridium;s_uncultured Clostridiales bacterium
k_Bacteria;p_Firmicutes;c_Clostridia;o_Clostridiales;f_Lachnospiraceae;g_Lachnospiraceae FCS020 group;s_unidentified
k_Bacteria;p_Firmicutes;c_Clostridia;o_Clostridiales;f_Lachnospiraceae;g_Lachnospiraceae NK4A136 group;NA
k_Bacteria;p_Firmicutes;c_Clostridia;o_Clostridiales;f_Lachnospiraceae;g_Lachnospiraceae NK4A136 group;s_uncultured bacterium
k_Bacteria;p_Firmicutes;c_Clostridia;o_Clostridiales;f_Lachnospiraceae;g_Lachnospiraceae NK4A136 group;s_uncultured Clostridiales bacterium
k_Bacteria;p_Firmicutes;c_Clostridia;o_Clostridiales;f_Lachnospiraceae;g_Lachnospiraceae NK4A136 group;s_unidentified
k_Bacteria;p_Firmicutes;c_Clostridia;o_Clostridiales;f_Lachnospiraceae;g_Lachnospiraceae UCG-001;NA
k_Bacteria;p_Firmicutes;c_Clostridia;o_Clostridiales;f_Lachnospiraceae;g_Lachnospiraceae UCG-001;s_uncultured bacterium
k_Bacteria;p_Firmicutes;c_Clostridia;o_Clostridiales;f_Lachnospiraceae;g_Lachnospiraceae UCG-001;s_uncultured Clostridiales bacterium
k_Bacteria;p_Firmicutes;c_Clostridia;o_Clostridiales;f_Lachnospiraceae;g_Lachnospiraceae UCG-004;NA
k_Bacteria;p_Firmicutes;c_Clostridia;o_Clostridiales;f_Lachnospiraceae;g_Lachnospiraceae UCG-004;s_uncultured organism
k_Bacteria;p_Firmicutes;c_Clostridia;o_Clostridiales;f_Lachnospiraceae;g_Lachnospiraceae UCG-005;NA
k_Bacteria;p_Firmicutes;c_Clostridia;o_Clostridiales;f_Lachnospiraceae;g_Lachnospiraceae UCG-005;s_uncultured organism
k_Bacteria;p_Firmicutes;c_Clostridia;o_Clostridiales;f_Lachnospiraceae;g_Lachnospiraceae UCG-005;s_unidentified
k_Bacteria;p_Firmicutes;c_Clostridia;o_Clostridiales;f_Lachnospiraceae;g_Lachnospiraceae UCG-006;s_uncultured bacterium
k_Bacteria;p_Firmicutes;c_Clostridia;o_Clostridiales;f_Lachnospiraceae;g_Lachnospiraceae UCG-008;s_uncultured Clostridiales bacterium
k_Bacteria;p_Firmicutes;c_Clostridia;o_Clostridiales;f_Lachnospiraceae;g_Lachnospiraceae UCG-010;s_uncultured bacterium
k_Bacteria;p_Firmicutes;c_Clostridia;o_Clostridiales;f_Lachnospiraceae;g_Marvinbryantia;s_uncultured bacterium
k_Bacteria;p_Firmicutes;c_Clostridia;o_Clostridiales;f_Lachnospiraceae;g_Roseburia;NA
k_Bacteria;p_Firmicutes;c_Clostridia;o_Clostridiales;f_Lachnospiraceae;g_Roseburia;s_Eubacterium sp
k_Bacteria;p_Firmicutes;c_Clostridia;o_Clostridiales;f_Lachnospiraceae;g_Roseburia;s_mouse gut metagenome
k_Bacteria;p_Firmicutes;c_Clostridia;o_Clostridiales;f_Lachnospiraceae;g_Roseburia;s_uncultured bacterium
k_Bacteria;p_Firmicutes;c_Clostridia;o_Clostridiales;f_Lachnospiraceae;g_Roseburia;s_uncultured Clostridiales bacterium
k_Bacteria;p_Firmicutes;c_Clostridia;o_Clostridiales;f_Lachnospiraceae;g_Roseburia;s_unidentified
k_Bacteria;p_Firmicutes;c_Clostridia;o_Clostridiales;f_Lachnospiraceae;g_Shuttleworthia;s_uncultured bacterium
k_Bacteria;p_Firmicutes;c_Clostridia;o_Clostridiales;f_Lachnospiraceae;g_Tyzzerella 3;s_uncultured bacterium
k_Bacteria;p_Firmicutes;c_Clostridia;o_Clostridiales;f_Lachnospiraceae;g_Tyzzerella;s_uncultured bacterium
k_Bacteria;p_Firmicutes;c_Clostridia;o_Clostridiales;f_Lachnospiraceae;g_uncultured;NA
k_Bacteria;p_Firmicutes;c_Clostridia;o_Clostridiales;f_Lachnospiraceae;g_uncultured;s_Clostridium sp
k_Bacteria;p_Firmicutes;c_Clostridia;o_Clostridiales;f_Lachnospiraceae;g_uncultured;s_Lachnospiraceae bacterium 3-1
k_Bacteria;p_Firmicutes;c_Clostridia;o_Clostridiales;f_Lachnospiraceae;g_uncultured;s_Lachnospiraceae bacterium 6-1
k_Bacteria;p_Firmicutes;c_Clostridia;o_Clostridiales;f_Lachnospiraceae;g_uncultured;s_mouse gut metagenome
k_Bacteria;p_Firmicutes;c_Clostridia;o_Clostridiales;f_Lachnospiraceae;g_uncultured;s_uncultured bacterium
k_Bacteria;p_Firmicutes;c_Clostridia;o_Clostridiales;f_Lachnospiraceae;g_uncultured;s_uncultured Clostridiales bacterium
k_Bacteria;p_Firmicutes;c_Clostridia;o_Clostridiales;f_Lachnospiraceae;g_uncultured;s_unidentified
k_Bacteria;p_Firmicutes;c_Clostridia;o_Clostridiales;f_Lachnospiraceae;NA;NA
k_Bacteria;p_Firmicutes;c_Clostridia;o_Clostridiales;f_Peptococcaceae;g_Peptococcus;s_uncultured bacterium
k_Bacteria;p_Firmicutes;c_Clostridia;o_Clostridiales;f_Peptococcaceae;g_uncultured;NA
k_Bacteria;p_Firmicutes;c_Clostridia;o_Clostridiales;f_Peptococcaceae;g_uncultured;s_unidentified
k_Bacteria;p_Firmicutes;c_Clostridia;o_Clostridiales;f_Ruminococcaceae;g_[Eubacterium] coprostanoligenes group;s_uncultured organism
k_Bacteria;p_Firmicutes;c_Clostridia;o_Clostridiales;f_Ruminococcaceae;g_Anaerotruncus;NA
k_Bacteria;p_Firmicutes;c_Clostridia;o_Clostridiales;f_Ruminococcaceae;g_Anaerotruncus;s_Anaerotruncus sp
k_Bacteria;p_Firmicutes;c_Clostridia;o_Clostridiales;f_Ruminococcaceae;g_Anaerotruncus;s_uncultured bacterium
k_Bacteria;p_Firmicutes;c_Clostridia;o_Clostridiales;f_Ruminococcaceae;g_Anaerotruncus;s_uncultured organism
k_Bacteria;p_Firmicutes;c_Clostridia;o_Clostridiales;f_Ruminococcaceae;g_Anaerotruncus;s_unidentified
k_Bacteria;p_Firmicutes;c_Clostridia;o_Clostridiales;f_Ruminococcaceae;g_Intestinimonas;NA
k_Bacteria;p_Firmicutes;c_Clostridia;o_Clostridiales;f_Ruminococcaceae;g_Intestinimonas;s_uncultured bacterium
k_Bacteria;p_Firmicutes;c_Clostridia;o_Clostridiales;f_Ruminococcaceae;g_Intestinimonas;s_uncultured Ruminococcaceae bacterium
k_Bacteria;p_Firmicutes;c_Clostridia;o_Clostridiales;f_Ruminococcaceae;g_Oscillibacter;NA
k_Bacteria;p_Firmicutes;c_Clostridia;o_Clostridiales;f_Ruminococcaceae;g_Oscillibacter;s_[Clostridium] leptum
k_Bacteria;p_Firmicutes;c_Clostridia;o_Clostridiales;f_Ruminococcaceae;g_Oscillibacter;s_uncultured bacterium
k_Bacteria;p_Firmicutes;c_Clostridia;o_Clostridiales;f_Ruminococcaceae;g_Oscillospira;NA

k_Bacteria;p_Firmicutes;c_Clostridia;o_Clostridiales;f_Ruminococcaceae;g_Ruminiclostridium 5;NA
k_Bacteria;p_Firmicutes;c_Clostridia;o_Clostridiales;f_Ruminococcaceae;g_Ruminiclostridium 5;s_uncultured bacterium
k_Bacteria;p_Firmicutes;c_Clostridia;o_Clostridiales;f_Ruminococcaceae;g_Ruminiclostridium 5;s_uncultured Clostridiales bacterium
k_Bacteria;p_Firmicutes;c_Clostridia;o_Clostridiales;f_Ruminococcaceae;g_Ruminiclostridium 5;s_unidentified
k_Bacteria;p_Firmicutes;c_Clostridia;o_Clostridiales;f_Ruminococcaceae;g_Ruminiclostridium 6;s_uncultured bacterium
k_Bacteria;p_Firmicutes;c_Clostridia;o_Clostridiales;f_Ruminococcaceae;g_Ruminiclostridium 9;NA
k_Bacteria;p_Firmicutes;c_Clostridia;o_Clostridiales;f_Ruminococcaceae;g_Ruminiclostridium 9;s_bacterium enrichment culture clone M244
k_Bacteria;p_Firmicutes;c_Clostridia;o_Clostridiales;f_Ruminococcaceae;g_Ruminiclostridium 9;s_uncultured bacterium
k_Bacteria;p_Firmicutes;c_Clostridia;o_Clostridiales;f_Ruminococcaceae;g_Ruminiclostridium 9;s_uncultured Clostridiales bacterium
k_Bacteria;p_Firmicutes;c_Clostridia;o_Clostridiales;f_Ruminococcaceae;g_Ruminiclostridium 9;s_unidentified
k_Bacteria;p_Firmicutes;c_Clostridia;o_Clostridiales;f_Ruminococcaceae;g_Ruminiclostridium;NA
k_Bacteria;p_Firmicutes;c_Clostridia;o_Clostridiales;f_Ruminococcaceae;g_Ruminiclostridium;s_bacterium NLAE-zl-H60
k_Bacteria;p_Firmicutes;c_Clostridia;o_Clostridiales;f_Ruminococcaceae;g_Ruminiclostridium;s_uncultured bacterium
k_Bacteria;p_Firmicutes;c_Clostridia;o_Clostridiales;f_Ruminococcaceae;g_Ruminococcaceae NK4A214 group;s_uncultured bacterium
k_Bacteria;p_Firmicutes;c_Clostridia;o_Clostridiales;f_Ruminococcaceae;g_Ruminococcaceae UCG-004;s_uncultured bacterium
k_Bacteria;p_Firmicutes;c_Clostridia;o_Clostridiales;f_Ruminococcaceae;g_Ruminococcaceae UCG-005;NA
k_Bacteria;p_Firmicutes;c_Clostridia;o_Clostridiales;f_Ruminococcaceae;g_Ruminococcaceae UCG-009;s_uncultured bacterium
k_Bacteria;p_Firmicutes;c_Clostridia;o_Clostridiales;f_Ruminococcaceae;g_Ruminococcaceae UCG-010;s_uncultured bacterium
k_Bacteria;p_Firmicutes;c_Clostridia;o_Clostridiales;f_Ruminococcaceae;g_Ruminococcaceae UCG-010;s_unidentified
k_Bacteria;p_Firmicutes;c_Clostridia;o_Clostridiales;f_Ruminococcaceae;g_Ruminococcaceae UCG-013;s_uncultured bacterium
k_Bacteria;p_Firmicutes;c_Clostridia;o_Clostridiales;f_Ruminococcaceae;g_Ruminococcaceae UCG-013;s_uncultured organism
k_Bacteria;p_Firmicutes;c_Clostridia;o_Clostridiales;f_Ruminococcaceae;g_Ruminococcaceae UCG-014;NA
k_Bacteria;p_Firmicutes;c_Clostridia;o_Clostridiales;f_Ruminococcaceae;g_Ruminococcaceae UCG-014;s_uncultured Acetivibrio sp
k_Bacteria;p_Firmicutes;c_Clostridia;o_Clostridiales;f_Ruminococcaceae;g_Ruminococcaceae UCG-014;s_uncultured bacterium
k_Bacteria;p_Firmicutes;c_Clostridia;o_Clostridiales;f_Ruminococcaceae;g_Ruminococcaceae UCG-014;s_uncultured Firmicutes bacterium
k_Bacteria;p_Firmicutes;c_Clostridia;o_Clostridiales;f_Ruminococcaceae;g_Ruminococcaceae UCG-014;s_uncultured rumen bacterium
k_Bacteria;p_Firmicutes;c_Clostridia;o_Clostridiales;f_Ruminococcaceae;g_Ruminococcaceae UCG-014;s_uncultured Ruminococcaceae bacterium
k_Bacteria;p_Firmicutes;c_Clostridia;o_Clostridiales;f_Ruminococcaceae;g_Ruminococcaceae UCG-014;s_unidentified
k_Bacteria;p_Firmicutes;c_Clostridia;o_Clostridiales;f_Ruminococcaceae;g_Ruminococcus 1;NA
k_Bacteria;p_Firmicutes;c_Clostridia;o_Clostridiales;f_Ruminococcaceae;g_Ruminococcus 1;s_uncultured bacterium
k_Bacteria;p_Firmicutes;c_Clostridia;o_Clostridiales;f_Ruminococcaceae;g_Ruminococcus 1;s_unidentified
k_Bacteria;p_Firmicutes;c_Clostridia;o_Clostridiales;f_Ruminococcaceae;g_uncultured;NA
k_Bacteria;p_Firmicutes;c_Clostridia;o_Clostridiales;f_Ruminococcaceae;g_uncultured;s_[Clostridium] leptum
k_Bacteria;p_Firmicutes;c_Clostridia;o_Clostridiales;f_Ruminococcaceae;g_uncultured;s_Oscillospiraceae bacterium VE202-24
k_Bacteria;p_Firmicutes;c_Clostridia;o_Clostridiales;f_Ruminococcaceae;g_uncultured;s_uncultured bacterium
k_Bacteria;p_Firmicutes;c_Clostridia;o_Clostridiales;f_Ruminococcaceae;g_uncultured;s_unidentified
k_Bacteria;p_Firmicutes;c_Clostridia;o_Clostridiales;f_Ruminococcaceae;NA;NA
k_Bacteria;p_Firmicutes;c_Clostridia;o_Clostridiales;NA;NA;NA
k_Bacteria;p_Firmicutes;c_Erysipelotrichia;o_Erysipelotrichales;f_Erysipelotrichaceae;g_uncultured;s_unidentified
k_Bacteria;p_Proteobacteria;c_Alphaproteobacteria;o_Rhodospirillales;f_Rhodospirillaceae;g_Thalassospira;s_Azospirillum sp
k_Bacteria;p_Proteobacteria;c_Alphaproteobacteria;o_Rickettsiales;f_Mitochondria;g_Oryza meyeriana;s_Oryza meyeriana
k_Bacteria;p_Proteobacteria;c_Alphaproteobacteria;o_Rickettsiales;f_Mitochondria;NA;NA
k_Bacteria;p_Proteobacteria;c_Betaproteobacteria;o_Burkholderiales;f_Alcaligenaceae;g_Parasutterella;s_uncultured bacterium
k_Bacteria;p_Proteobacteria;c_Deltaproteobacteria;o_Desulfovibrionales;f_Desulfovibrionaceae;g_Bilophila;s_uncultured bacterium
k_Bacteria;p_Proteobacteria;c_Deltaproteobacteria;o_Desulfovibrionales;f_Desulfovibrionaceae;g_Desulfovibrio;s_unidentified
k_Bacteria;p_Proteobacteria;c_Epsilonproteobacteria;o_Campylobacterales;f_Helicobacteraceae;g_Helicobacter;NA
k_Bacteria;p_Proteobacteria;c_Epsilonproteobacteria;o_Campylobacterales;f_Helicobacteraceae;g_Helicobacter;s_uncultured Helicobacter sp
k_Bacteria;p_Saccharibacteria;c_Unknown Class;o_Unknown Order;f_Unknown Family;g_Candidatus Saccharimonas;s_uncultured bacterium
k_Bacteria;p_Tenericutes;c_Mollicutes;o_Anaeroplasmatales;f_Anaeroplasmataceae;g_Anaeroplasma;s_uncultured organism
k_Bacteria;p_Tenericutes;c_Mollicutes;o_Anaeroplasmatales;f_Anaeroplasmataceae;g_Anaeroplasma;s_unidentified
k_Bacteria;p_Tenericutes;c_Mollicutes;o_Mollicutes RF9;f_uncultured bacterium;g_uncultured bacterium;s_uncultured bacterium
k_Bacteria;p_Tenericutes;c_Mollicutes;o_Mollicutes RF9;f_unidentified;g_unidentified;s_unidentified
k_Bacteria;p_Tenericutes;c_Mollicutes;o_Mollicutes RF9;NA;NA;NA
NA;NA;NA;NA;NA;NA;NA
NA;p_Firmicutes;c_Clostridia;o_Clostridiales;f_Lachnospiraceae;NA;NA

Legend for relative abundance at species level (Supplementary Figure 6) CIA samples

k_Bacteria;p_Actinobacteria;c_Coriobacteriia;o_Coriobacteriales;f_Coriobacteriaceae;g_Enterorhabdus;s_Enterorhabdus mucosicola
k_Bacteria;p_Actinobacteria;c_Coriobacteriia;o_Coriobacteriales;f_Coriobacteriaceae;g_Enterorhabdus;s_mouse gut metagenome
k_Bacteria;p_Actinobacteria;c_Coriobacteriia;o_Coriobacteriales;f_Coriobacteriaceae;g_Enterorhabdus;s_uncultured bacterium
k_Bacteria;p_Actinobacteria;c_Coriobacteriia;o_Coriobacteriales;f_Coriobacteriaceae;g_Enterorhabdus;s_unidentified
k_Bacteria;p_Actinobacteria;c_Coriobacteriia;o_Coriobacteriales;f_Coriobacteriaceae;g_uncultured;s_uncultured Coriobacteriales bacterium
k_Bacteria;p_Actinobacteria;c_Coriobacteriia;o_Coriobacteriales;f_Coriobacteriaceae;NA;NA
k_Bacteria;p_Bacteroidetes;c_Bacteroidia;o_Bacteroidales;f_Bacteroidaceae;g_Bacteroides;NA
k_Bacteria;p_Bacteroidetes;c_Bacteroidia;o_Bacteroidales;f_Bacteroidaceae;g_Bacteroides;s_Bacteroides acidifaciens
k_Bacteria;p_Bacteroidetes;c_Bacteroidia;o_Bacteroidales;f_Bacteroidaceae;g_Bacteroides;s_human gut metagenome
k_Bacteria;p_Bacteroidetes;c_Bacteroidia;o_Bacteroidales;f_Bacteroidaceae;g_Bacteroides;s_unidentified
k_Bacteria;p_Bacteroidetes;c_Bacteroidia;o_Bacteroidales;f_Bacteroidales S24-7 group;g_mouse gut metagenome;s_mouse gut metagenome
k_Bacteria;p_Bacteroidetes;c_Bacteroidia;o_Bacteroidales;f_Bacteroidales S24-7 group;g_uncultured bacterium;s_uncultured bacterium
k_Bacteria;p_Bacteroidetes;c_Bacteroidia;o_Bacteroidales;f_Bacteroidales S24-7 group;g_uncultured Bacteroidales bacterium;s_uncultured Bacteroidales bacterium
k_Bacteria;p_Bacteroidetes;c_Bacteroidia;o_Bacteroidales;f_Bacteroidales S24-7 group;g_uncultured organism;s_uncultured organism
k_Bacteria;p_Bacteroidetes;c_Bacteroidia;o_Bacteroidales;f_Bacteroidales S24-7 group;NA;NA
k_Bacteria;p_Bacteroidetes;c_Bacteroidia;o_Bacteroidales;f_Porphyromonadaceae;g_Odoribacter;s_uncultured bacterium
k_Bacteria;p_Bacteroidetes;c_Bacteroidia;o_Bacteroidales;f_Porphyromonadaceae;g_Odoribacter;s_unidentified
k_Bacteria;p_Bacteroidetes;c_Bacteroidia;o_Bacteroidales;f_Porphyromonadaceae;g_Parabacteroides;NA
k_Bacteria;p_Bacteroidetes;c_Bacteroidia;o_Bacteroidales;f_Porphyromonadaceae;g_Parabacteroides;s_Parabacteroides goldsteinii
k_Bacteria;p_Bacteroidetes;c_Bacteroidia;o_Bacteroidales;f_Prevotellaceae;g_Prevotellaceae UCG-001;s_uncultured bacterium
k_Bacteria;p_Bacteroidetes;c_Bacteroidia;o_Bacteroidales;f_Rikenellaceae;g_Alistipes;NA
k_Bacteria;p_Bacteroidetes;c_Bacteroidia;o_Bacteroidales;f_Rikenellaceae;g_Alistipes;s_Bacteroidales bacterium ph8
k_Bacteria;p_Bacteroidetes;c_Bacteroidia;o_Bacteroidales;f_Rikenellaceae;g_Alistipes;s_uncultured bacterium
k_Bacteria;p_Bacteroidetes;c_Bacteroidia;o_Bacteroidales;f_Rikenellaceae;g_Alistipes;s_uncultured organism
k_Bacteria;p_Bacteroidetes;c_Bacteroidia;o_Bacteroidales;f_Rikenellaceae;g_Alistipes;s_unidentified
k_Bacteria;p_Bacteroidetes;c_Bacteroidia;o_Bacteroidales;f_Rikenellaceae;g_Rikenella;s_uncultured bacterium
k_Bacteria;p_Bacteroidetes;c_Bacteroidia;o_Bacteroidales;f_Rikenellaceae;g_Rikenellaceae RC9 gut group;s_uncultured bacterium
k_Bacteria;p_Cyanobacteria;c_Chloroplast;o_Phaseolus acutifolius (tepary bean);f_Phaseolus acutifolius (tepary bean);g_Phaseolus acutifolius (tepary bean);s_Phaseolus acutifolius (tepary bean)
k_Bacteria;p_Deferribacteres;c_Deferribacteres;o_Deferribacterales;f_Deferribacteraceae;g_Mucispirillum;NA
k_Bacteria;p_Deferribacteres;c_Deferribacteres;o_Deferribacterales;f_Deferribacteraceae;g_Mucispirillum;s_uncultured bacterium
k_Bacteria;p_Firmicutes;c_Bacilli;o_Lactobacillales;f_Enterococcaceae;g_Enterococcus;NA
k_Bacteria;p_Firmicutes;c_Bacilli;o_Lactobacillales;f_Lactobacillaceae;g_Lactobacillus;s_Lactobacillus faecis
k_Bacteria;p_Firmicutes;c_Bacilli;o_Lactobacillales;f_Lactobacillaceae;g_Lactobacillus;s_Lactobacillus gasseri
k_Bacteria;p_Firmicutes;c_Bacilli;o_Lactobacillales;f_Lactobacillaceae;g_Lactobacillus;s_Lactobacillus intestinalis
k_Bacteria;p_Firmicutes;c_Bacilli;o_Lactobacillales;f_Lactobacillaceae;g_Lactobacillus;s_Lactobacillus johnsonii
k_Bacteria;p_Firmicutes;c_Bacilli;o_Lactobacillales;f_Lactobacillaceae;g_Lactobacillus;s_Lactobacillus reuteri
k_Bacteria;p_Firmicutes;c_Bacilli;o_Lactobacillales;f_Lactobacillaceae;g_Lactobacillus;s_Lactobacillus salivarius
k_Bacteria;p_Firmicutes;c_Bacilli;o_Lactobacillales;f_Streptococcaceae;g_Streptococcus;s_Streptococcus danieliae
k_Bacteria;p_Firmicutes;c_Clostridia;o_Clostridiales;f_Clostridiales vadinBB60 group;g_Clostridiales bacterium JN18_A56_K;s_Clostridiales bacterium JN18_A56_K
k_Bacteria;p_Firmicutes;c_Clostridia;o_Clostridiales;f_Clostridiales vadinBB60 group;g_uncultured bacterium;s_uncultured bacterium
k_Bacteria;p_Firmicutes;c_Clostridia;o_Clostridiales;f_Clostridiales vadinBB60 group;g_unidentified;s_unidentified
k_Bacteria;p_Firmicutes;c_Clostridia;o_Clostridiales;f_Clostridiales vadinBB60 group;NA;NA
k_Bacteria;p_Firmicutes;c_Clostridia;o_Clostridiales;f_Family XIII;g_[Eubacterium] brachy group;s_uncultured bacterium
k_Bacteria;p_Firmicutes;c_Clostridia;o_Clostridiales;f_Family XIII;g_[Eubacterium] nodatum group;s_uncultured organism
k_Bacteria;p_Firmicutes;c_Clostridia;o_Clostridiales;f_Family XIII;g_Family XIII AD3011 group;s_uncultured bacterium
k_Bacteria;p_Firmicutes;c_Clostridia;o_Clostridiales;f_Family XIII;g_Family XIII UCG-001;s_uncultured bacterium
k_Bacteria;p_Firmicutes;c_Clostridia;o_Clostridiales;f_Lachnospiraceae;g_Acetatifactor;NA
k_Bacteria;p_Firmicutes;c_Clostridia;o_Clostridiales;f_Lachnospiraceae;g_Acetatifactor;s_uncultured bacterium
k_Bacteria;p_Firmicutes;c_Clostridia;o_Clostridiales;f_Lachnospiraceae;g_Blautia;NA
k_Bacteria;p_Firmicutes;c_Clostridia;o_Clostridiales;f_Lachnospiraceae;g_Blautia;s_Lachnospiraceae bacterium 615
k_Bacteria;p_Firmicutes;c_Clostridia;o_Clostridiales;f_Lachnospiraceae;g_Coprococcus 1;s_uncultured bacterium
k_Bacteria;p_Firmicutes;c_Clostridia;o_Clostridiales;f_Lachnospiraceae;g_Coprococcus 1;s_unidentified
k_Bacteria;p_Firmicutes;c_Clostridia;o_Clostridiales;f_Lachnospiraceae;g_Incertae Sedis;s_uncultured bacterium
k_Bacteria;p_Firmicutes;c_Clostridia;o_Clostridiales;f_Lachnospiraceae;g_Lachnoclostridium;NA
k_Bacteria;p_Firmicutes;c_Clostridia;o_Clostridiales;f_Lachnospiraceae;g_Lachnoclostridium;s_[Clostridium] scindens
k_Bacteria;p_Firmicutes;c_Clostridia;o_Clostridiales;f_Lachnospiraceae;g_Lachnoclostridium;s_uncultured bacterium
k_Bacteria;p_Firmicutes;c_Clostridia;o_Clostridiales;f_Lachnospiraceae;g_Lachnoclostridium;s_uncultured Clostridiales bacterium
k_Bacteria;p_Firmicutes;c_Clostridia;o_Clostridiales;f_Lachnospiraceae;g_Lachnospiraceae FCS020 group;s_unidentified
k_Bacteria;p_Firmicutes;c_Clostridia;o_Clostridiales;f_Lachnospiraceae;g_Lachnospiraceae NK4A136 group;NA
k_Bacteria;p_Firmicutes;c_Clostridia;o_Clostridiales;f_Lachnospiraceae;g_Lachnospiraceae NK4A136 group;s_uncultured bacterium
k_Bacteria;p_Firmicutes;c_Clostridia;o_Clostridiales;f_Lachnospiraceae;g_Lachnospiraceae NK4A136 group;s_uncultured Clostridiales bacterium
k_Bacteria;p_Firmicutes;c_Clostridia;o_Clostridiales;f_Lachnospiraceae;g_Lachnospiraceae NK4A136 group;s_unidentified
k_Bacteria;p_Firmicutes;c_Clostridia;o_Clostridiales;f_Lachnospiraceae;g_Lachnospiraceae UCG-001;NA
k_Bacteria;p_Firmicutes;c_Clostridia;o_Clostridiales;f_Lachnospiraceae;g_Lachnospiraceae UCG-001;s_uncultured bacterium
k_Bacteria;p_Firmicutes;c_Clostridia;o_Clostridiales;f_Lachnospiraceae;g_Lachnospiraceae UCG-001;s_uncultured Clostridiales bacterium
k_Bacteria;p_Firmicutes;c_Clostridia;o_Clostridiales;f_Lachnospiraceae;g_Lachnospiraceae UCG-004;NA
k_Bacteria;p_Firmicutes;c_Clostridia;o_Clostridiales;f_Lachnospiraceae;g_Lachnospiraceae UCG-004;s_uncultured organism
k_Bacteria;p_Firmicutes;c_Clostridia;o_Clostridiales;f_Lachnospiraceae;g_Lachnospiraceae UCG-005;NA
k_Bacteria;p_Firmicutes;c_Clostridia;o_Clostridiales;f_Lachnospiraceae;g_Lachnospiraceae UCG-005;s_uncultured organism
k_Bacteria;p_Firmicutes;c_Clostridia;o_Clostridiales;f_Lachnospiraceae;g_Lachnospiraceae UCG-005;s_unidentified
k_Bacteria;p_Firmicutes;c_Clostridia;o_Clostridiales;f_Lachnospiraceae;g_Lachnospiraceae UCG-006;s_uncultured bacterium
k_Bacteria;p_Firmicutes;c_Clostridia;o_Clostridiales;f_Lachnospiraceae;g_Lachnospiraceae UCG-008;s_uncultured Clostridiales bacterium
k_Bacteria;p_Firmicutes;c_Clostridia;o_Clostridiales;f_Lachnospiraceae;g_Lachnospiraceae UCG-010;s_uncultured bacterium
k_Bacteria;p_Firmicutes;c_Clostridia;o_Clostridiales;f_Lachnospiraceae;g_Marvinbryantia;s_uncultured bacterium
k_Bacteria;p_Firmicutes;c_Clostridia;o_Clostridiales;f_Lachnospiraceae;g_Roseburia;NA
k_Bacteria;p_Firmicutes;c_Clostridia;o_Clostridiales;f_Lachnospiraceae;g_Roseburia;s_Eubacterium sp
k_Bacteria;p_Firmicutes;c_Clostridia;o_Clostridiales;f_Lachnospiraceae;g_Roseburia;s_mouse gut metagenome
k_Bacteria;p_Firmicutes;c_Clostridia;o_Clostridiales;f_Lachnospiraceae;g_Roseburia;s_uncultured bacterium
k_Bacteria;p_Firmicutes;c_Clostridia;o_Clostridiales;f_Lachnospiraceae;g_Roseburia;s_uncultured Clostridiales bacterium
k_Bacteria;p_Firmicutes;c_Clostridia;o_Clostridiales;f_Lachnospiraceae;g_Roseburia;s_unidentified
k_Bacteria;p_Firmicutes;c_Clostridia;o_Clostridiales;f_Lachnospiraceae;g_Shuttleworthia;s_uncultured bacterium
k_Bacteria;p_Firmicutes;c_Clostridia;o_Clostridiales;f_Lachnospiraceae;g_Tyzzerella 3;s_uncultured bacterium
k_Bacteria;p_Firmicutes;c_Clostridia;o_Clostridiales;f_Lachnospiraceae;g_Tyzzerella;s_uncultured bacterium
k_Bacteria;p_Firmicutes;c_Clostridia;o_Clostridiales;f_Lachnospiraceae;g_uncultured;NA
k_Bacteria;p_Firmicutes;c_Clostridia;o_Clostridiales;f_Lachnospiraceae;g_uncultured;s_Clostridium sp
k_Bacteria;p_Firmicutes;c_Clostridia;o_Clostridiales;f_Lachnospiraceae;g_uncultured;s_Lachnospiraceae bacterium 3-1
k_Bacteria;p_Firmicutes;c_Clostridia;o_Clostridiales;f_Lachnospiraceae;g_uncultured;s_Lachnospiraceae bacterium 6-1
k_Bacteria;p_Firmicutes;c_Clostridia;o_Clostridiales;f_Lachnospiraceae;g_uncultured;s_mouse gut metagenome
k_Bacteria;p_Firmicutes;c_Clostridia;o_Clostridiales;f_Lachnospiraceae;g_uncultured;s_uncultured bacterium
k_Bacteria;p_Firmicutes;c_Clostridia;o_Clostridiales;f_Lachnospiraceae;g_uncultured;s_uncultured Clostridiales bacterium
k_Bacteria;p_Firmicutes;c_Clostridia;o_Clostridiales;f_Lachnospiraceae;g_uncultured;s_unidentified
k_Bacteria;p_Firmicutes;c_Clostridia;o_Clostridiales;f_Lachnospiraceae;NA;NA
k_Bacteria;p_Firmicutes;c_Clostridia;o_Clostridiales;f_Peptococcaceae;g_Peptococcus;s_uncultured bacterium
k_Bacteria;p_Firmicutes;c_Clostridia;o_Clostridiales;f_Peptococcaceae;g_uncultured;NA
k_Bacteria;p_Firmicutes;c_Clostridia;o_Clostridiales;f_Peptococcaceae;g_uncultured;s_unidentified
k_Bacteria;p_Firmicutes;c_Clostridia;o_Clostridiales;f_Ruminococcaceae;g_[Eubacterium] coprostanoligenes group;s_uncultured organism
k_Bacteria;p_Firmicutes;c_Clostridia;o_Clostridiales;f_Ruminococcaceae;g_Anaerotruncus;NA
k_Bacteria;p_Firmicutes;c_Clostridia;o_Clostridiales;f_Ruminococcaceae;g_Anaerotruncus;s_Anaerotruncus sp
k_Bacteria;p_Firmicutes;c_Clostridia;o_Clostridiales;f_Ruminococcaceae;g_Anaerotruncus;s_uncultured bacterium
k_Bacteria;p_Firmicutes;c_Clostridia;o_Clostridiales;f_Ruminococcaceae;g_Anaerotruncus;s_uncultured organism
k_Bacteria;p_Firmicutes;c_Clostridia;o_Clostridiales;f_Ruminococcaceae;g_Anaerotruncus;s_unidentified
k_Bacteria;p_Firmicutes;c_Clostridia;o_Clostridiales;f_Ruminococcaceae;g_Intestinimonas;NA
k_Bacteria;p_Firmicutes;c_Clostridia;o_Clostridiales;f_Ruminococcaceae;g_Intestinimonas;s_uncultured bacterium
k_Bacteria;p_Firmicutes;c_Clostridia;o_Clostridiales;f_Ruminococcaceae;g_Intestinimonas;s_uncultured Ruminococcaceae bacterium
k_Bacteria;p_Firmicutes;c_Clostridia;o_Clostridiales;f_Ruminococcaceae;g_Oscillibacter;NA
k_Bacteria;p_Firmicutes;c_Clostridia;o_Clostridiales;f_Ruminococcaceae;g_Oscillibacter;s_[Clostridium] leptum
k_Bacteria;p_Firmicutes;c_Clostridia;o_Clostridiales;f_Ruminococcaceae;g_Oscillibacter;s_uncultured bacterium
k_Bacteria;p_Firmicutes;c_Clostridia;o_Clostridiales;f_Ruminococcaceae;g_Oscillospira;NA

k_Bacteria;p_Firmicutes;c_Clostridia;o_Clostridiales;f_Ruminococcaceae;g_Ruminiclostridium 5;NA
k_Bacteria;p_Firmicutes;c_Clostridia;o_Clostridiales;f_Ruminococcaceae;g_Ruminiclostridium 5;s_uncultured bacterium
k_Bacteria;p_Firmicutes;c_Clostridia;o_Clostridiales;f_Ruminococcaceae;g_Ruminiclostridium 5;s_uncultured Clostridiales bacterium
k_Bacteria;p_Firmicutes;c_Clostridia;o_Clostridiales;f_Ruminococcaceae;g_Ruminiclostridium 5;s_unidentified
k_Bacteria;p_Firmicutes;c_Clostridia;o_Clostridiales;f_Ruminococcaceae;g_Ruminiclostridium 6;s_uncultured bacterium
k_Bacteria;p_Firmicutes;c_Clostridia;o_Clostridiales;f_Ruminococcaceae;g_Ruminiclostridium 9;NA
k_Bacteria;p_Firmicutes;c_Clostridia;o_Clostridiales;f_Ruminococcaceae;g_Ruminiclostridium 9;s_bacterium enrichment culture clone M244
k_Bacteria;p_Firmicutes;c_Clostridia;o_Clostridiales;f_Ruminococcaceae;g_Ruminiclostridium 9;s_uncultured bacterium
k_Bacteria;p_Firmicutes;c_Clostridia;o_Clostridiales;f_Ruminococcaceae;g_Ruminiclostridium 9;s_uncultured Clostridiales bacterium
k_Bacteria;p_Firmicutes;c_Clostridia;o_Clostridiales;f_Ruminococcaceae;g_Ruminiclostridium 9;s_unidentified
k_Bacteria;p_Firmicutes;c_Clostridia;o_Clostridiales;f_Ruminococcaceae;g_Ruminiclostridium;NA
k_Bacteria;p_Firmicutes;c_Clostridia;o_Clostridiales;f_Ruminococcaceae;g_Ruminiclostridium;s_bacterium NLAE-zl-H60
k_Bacteria;p_Firmicutes;c_Clostridia;o_Clostridiales;f_Ruminococcaceae;g_Ruminiclostridium;s_uncultured bacterium
k_Bacteria;p_Firmicutes;c_Clostridia;o_Clostridiales;f_Ruminococcaceae;g_Ruminococcaceae NK4A214 group;s_uncultured bacterium
k_Bacteria;p_Firmicutes;c_Clostridia;o_Clostridiales;f_Ruminococcaceae;g_Ruminococcaceae UCG-004;s_uncultured bacterium
k_Bacteria;p_Firmicutes;c_Clostridia;o_Clostridiales;f_Ruminococcaceae;g_Ruminococcaceae UCG-005;NA
k_Bacteria;p_Firmicutes;c_Clostridia;o_Clostridiales;f_Ruminococcaceae;g_Ruminococcaceae UCG-009;s_uncultured bacterium
k_Bacteria;p_Firmicutes;c_Clostridia;o_Clostridiales;f_Ruminococcaceae;g_Ruminococcaceae UCG-010;s_uncultured bacterium
k_Bacteria;p_Firmicutes;c_Clostridia;o_Clostridiales;f_Ruminococcaceae;g_Ruminococcaceae UCG-010;s_unidentified
k_Bacteria;p_Firmicutes;c_Clostridia;o_Clostridiales;f_Ruminococcaceae;g_Ruminococcaceae UCG-013;s_uncultured bacterium
k_Bacteria;p_Firmicutes;c_Clostridia;o_Clostridiales;f_Ruminococcaceae;g_Ruminococcaceae UCG-013;s_uncultured organism
k_Bacteria;p_Firmicutes;c_Clostridia;o_Clostridiales;f_Ruminococcaceae;g_Ruminococcaceae UCG-014;NA
k_Bacteria;p_Firmicutes;c_Clostridia;o_Clostridiales;f_Ruminococcaceae;g_Ruminococcaceae UCG-014;s_uncultured Acetivibrio sp
k_Bacteria;p_Firmicutes;c_Clostridia;o_Clostridiales;f_Ruminococcaceae;g_Ruminococcaceae UCG-014;s_uncultured bacterium
k_Bacteria;p_Firmicutes;c_Clostridia;o_Clostridiales;f_Ruminococcaceae;g_Ruminococcaceae UCG-014;s_uncultured Firmicutes bacterium
k_Bacteria;p_Firmicutes;c_Clostridia;o_Clostridiales;f_Ruminococcaceae;g_Ruminococcaceae UCG-014;s_uncultured rumen bacterium
k_Bacteria;p_Firmicutes;c_Clostridia;o_Clostridiales;f_Ruminococcaceae;g_Ruminococcaceae UCG-014;s_uncultured Ruminococcaceae bacterium
k_Bacteria;p_Firmicutes;c_Clostridia;o_Clostridiales;f_Ruminococcaceae;g_Ruminococcaceae UCG-014;s_unidentified
k_Bacteria;p_Firmicutes;c_Clostridia;o_Clostridiales;f_Ruminococcaceae;g_Ruminococcus 1;NA
k_Bacteria;p_Firmicutes;c_Clostridia;o_Clostridiales;f_Ruminococcaceae;g_Ruminococcus 1;s_uncultured bacterium
k_Bacteria;p_Firmicutes;c_Clostridia;o_Clostridiales;f_Ruminococcaceae;g_Ruminococcus 1;s_unidentified
k_Bacteria;p_Firmicutes;c_Clostridia;o_Clostridiales;f_Ruminococcaceae;g_uncultured;NA
k_Bacteria;p_Firmicutes;c_Clostridia;o_Clostridiales;f_Ruminococcaceae;g_uncultured;s_[Clostridium] leptum
k_Bacteria;p_Firmicutes;c_Clostridia;o_Clostridiales;f_Ruminococcaceae;g_uncultured;s_Oscillospiraceae bacterium VE202-24
k_Bacteria;p_Firmicutes;c_Clostridia;o_Clostridiales;f_Ruminococcaceae;g_uncultured;s_uncultured bacterium
k_Bacteria;p_Firmicutes;c_Clostridia;o_Clostridiales;f_Ruminococcaceae;g_uncultured;s_unidentified
k_Bacteria;p_Firmicutes;c_Clostridia;o_Clostridiales;f_Ruminococcaceae;NA;NA
k_Bacteria;p_Firmicutes;c_Clostridia;o_Clostridiales;NA;NA;NA
k_Bacteria;p_Firmicutes;c_Erysipelotrichia;o_Erysipelotrichales;f_Erysipelotrichaceae;g_uncultured;s_unidentified
k_Bacteria;p_Proteobacteria;c_Alphaproteobacteria;o_Rhodospirillales;f_Rhodospirillaceae;g_Thalassospira;s_Azospirillum sp
k_Bacteria;p_Proteobacteria;c_Alphaproteobacteria;o_Rickettsiales;f_Mitochondria;g_Oryza meyeriana;s_Oryza meyeriana
k_Bacteria;p_Proteobacteria;c_Alphaproteobacteria;o_Rickettsiales;f_Mitochondria;NA;NA
k_Bacteria;p_Proteobacteria;c_Betaproteobacteria;o_Burkholderiales;f_Alcaligenaceae;g_Parasutterella;s_uncultured bacterium
k_Bacteria;p_Proteobacteria;c_Deltaproteobacteria;o_Desulfovibrionales;f_Desulfovibrionaceae;g_Bilophila;s_uncultured bacterium
k_Bacteria;p_Proteobacteria;c_Deltaproteobacteria;o_Desulfovibrionales;f_Desulfovibrionaceae;g_Desulfovibrio;s_unidentified
k_Bacteria;p_Proteobacteria;c_Epsilonproteobacteria;o_Campylobacterales;f_Helicobacteraceae;g_Helicobacter;NA
k_Bacteria;p_Proteobacteria;c_Epsilonproteobacteria;o_Campylobacterales;f_Helicobacteraceae;g_Helicobacter;s_uncultured Helicobacter sp
k_Bacteria;p_Saccharibacteria;c_Unknown Class;o_Unknown Order;f_Unknown Family;g_Candidatus Saccharimonas;s_uncultured bacterium
k_Bacteria;p_Tenericutes;c_Mollicutes;o_Anaeroplasmatales;f_Anaeroplasmataceae;g_Anaeroplasma;s_uncultured organism
k_Bacteria;p_Tenericutes;c_Mollicutes;o_Anaeroplasmatales;f_Anaeroplasmataceae;g_Anaeroplasma;s_unidentified
k_Bacteria;p_Tenericutes;c_Mollicutes;o_Mollicutes RF9;f_uncultured bacterium;g_uncultured bacterium;s_uncultured bacterium
k_Bacteria;p_Tenericutes;c_Mollicutes;o_Mollicutes RF9;f_unidentified;g_unidentified;s_unidentified
k_Bacteria;p_Tenericutes;c_Mollicutes;o_Mollicutes RF9;NA;NA;NA
NA;NA;NA;NA;NA;NA;NA
NA;p_Firmicutes;c_Clostridia;o_Clostridiales;f_Lachnospiraceae;NA;NA

Supplementary Figure 8: Legend for relative abundance at species level plots in Supplementary Figure 6.
